# Sparsely Distributed, Pre-synaptic Kv3 K^+^ Channels Control Spontaneous Firing and Cross-Unit Synchrony via the Regulation of Synaptic Noise in an Auditory Brainstem Circuit

**DOI:** 10.3389/fncel.2021.721371

**Published:** 2021-09-03

**Authors:** Timothy OIsen, Alberto Capurro, Maša Švent, Nadia Pilati, Charles Large, Nick Hartell, Martine Hamann

**Affiliations:** ^1^Department of Neuroscience, Psychology and Behaviour, College of Life Sciences, University of Leicester, Leicester, United Kingdom; ^2^Department of Otolaryngology-Head and Neck Surgery, University of California, San Francisco, San Francisco, CA, United States; ^3^Biosciences Institute, Newcastle University, Newcastle upon Tyne, United Kingdom; ^4^Autifony Srl, Padua, Italy; ^5^Autifony Therapeutics Limited, Stevenage Bioscience Catalyst, Stevenage, United Kingdom

**Keywords:** Kv3 K^+^ currents, synaptic release, excitability, auditory brainstem, pre-synaptic, synchrony, calcium imaging, noise

## Abstract

Spontaneous subthreshold activity in the central nervous system is fundamental to information processing and transmission, as it amplifies and optimizes sub-threshold signals, thereby improving action potential initiation and maintaining reliable firing. This form of spontaneous activity, which is frequently considered noise, is particularly important at auditory synapses where acoustic information is encoded by rapid and temporally precise firing rates. In contrast, when present in excess, this form of noise becomes detrimental to acoustic information as it contributes to the generation and maintenance of auditory disorders such as tinnitus. The most prominent contribution to subthreshold noise is spontaneous synaptic transmission (synaptic noise). Although numerous studies have examined the role of synaptic noise on single cell excitability, little is known about its pre-synaptic modulation owing in part to the difficulties of combining noise modulation with monitoring synaptic release. Here we study synaptic noise in the auditory brainstem dorsal cochlear nucleus (DCN) of mice and show that pharmacological potentiation of Kv3 K^+^ currents reduces the level of synaptic bombardment onto DCN principal fusiform cells. Using a transgenic mouse line (SyG37) expressing SyGCaMP2-mCherry, a calcium sensor that targets pre-synaptic terminals, we show that positive Kv3 K^+^ current modulation decreases calcium influx in a fifth of pre-synaptic boutons. Furthermore, while maintaining rapid and precise spike timing, positive Kv3 K^+^ current modulation increases the synchronization of local circuit neurons by reducing spontaneous activity. In conclusion, our study identifies a unique pre-synaptic mechanism which reduces synaptic noise at auditory synapses and contributes to the coherent activation of neurons in a local auditory brainstem circuit. This form of modulation highlights a new therapeutic target, namely the pre-synaptic bouton, for ameliorating the effects of hearing disorders which are dependent on aberrant spontaneous activity within the central auditory system.

## Introduction

Biological noise is ubiquitous throughout sensory systems and enhances the detection of weak signals via stochastic resonance-based mechanisms (Longtin, 1993; Wiesenfeld and Moss, 1995; Collins et al., 1997; Simonotto et al., 1997; Jaramillo and Wiesenfeld, 1998; Moss et al., 2004; Krauss et al., 2018). In the visual system, noise enhances edge detection (Simonotto et al., 1997), improves the discrimination of visual motion (Treviño et al., 2016) and facilitates the discrimination of objects in visually impaired patients (Itzcovich et al., 2017). Noise also improves auditory processing as it enhances the neural encoding of vowels (Morse and Evans, 1996), decreases auditory thresholds (Zeng et al., 2000), enhances pitch sensation (Martignoli et al., 2013), and improves the sensitivity of cochlear implants (Chatterjee and Robert, 2001; Chatterjee and Oba, 2005) and thus helps in the restoration of auditory processing following hearing degradation. However, when present in excess, noise can distort incoming sensory signals (Collins et al., 1997; Simonotto et al., 1997; Bidelman, 2017; Bidelman et al., 2020). As biological noise is likely to be beneficial within certain limit boundaries, it is important to understand mechanisms involved in its modulation.

Spontaneous synaptic transmission arising from the spontaneous release of synaptic vesicles, otherwise known as synaptic noise, is a key source of intrinsic biological noise in the CNS (Faisal et al., 2008). Synaptic noise improves the fidelity of synaptic transmission (Stacey and Durand, 2001; Sedigh-Sarvestani et al., 2019). However, an excess of synaptic noise in the auditory system is associated with the perception of tinnitus (Yang et al., 2011). Despite a growing body of research demonstrating the involvement of pre-synaptic ion channels in the regulation of synaptic transmission (Dodson and Forsythe, 2004; Engel and Jonas, 2005; Alle et al., 2011; Rowan et al., 2016; Burke and Bender, 2019), little is known about the pre-synaptic role of those channels in regulating general levels of excitability and action potential firing rate. Voltage-activated K^+^ channels (Kv) are known to have a major role in the regulation of network-level excitability in the central auditory system (Song et al., 2005; Steinert et al., 2011; Pilati et al., 2012; Li et al., 2015), but the pre-synaptic contribution of Kv channels to excitability remains relatively unstudied.

The dorsal cochlear nucleus (DCN) is a structure in the auditory brainstem that is essential for the encoding of vertical sound localization and the integration of auditory with somatosensory signals (Tzounopoulos et al., 2004, 2007; Reiss and Young, 2005; Tzounopoulos and Kraus, 2009; Koehler and Shore, 2013). Dysfunction of synaptic transmission within this structure is associated with hyperexcitable action potential firing and the induction of tinnitus (Zeng et al., 2009; Middleton et al., 2011; Shore, 2011). Our previous study showed that potentiating Kv3 K^+^ currents using AUT1, a Kv3 K^+^ channel positive modulator (Rosato-Siri et al., 2015; Brown et al., 2016), reduced synaptic noise and firing rates at DCN principal fusiform cells (Olsen et al., 2018). Using a ratiometric calcium sensor SyGCaMP2-mCherry, a calcium sensor that is restricted to pre-synaptic boutons (Al-Osta et al., 2018), we are here studying the pre-synaptic role of Kv3 K^+^ channels on the action potential firing rate in the DCN. We demonstrate that Kv3 K^+^ channels are sparsely localized to a proportion of pre-synaptic boutons in the DCN, and that their increased activation reduces the level of synaptic noise and the spontaneous firing rates of DCN principal fusiform cells. We also studied DCN fusiform cell firing rates in relation to the general excitability monitored in the fusiform cell layer and demonstrate that increasing Kv3 K^+^ channel activation increases the synchronized firing between fusiform and their neighboring cells.

## Materials and Methods

Experiments were carried out in accordance with the United Kingdom Animals (Scientific Procedures) Act of 1986 and approved by the Home Office and Leicester University Ethical Committee.

### Electrophysiological Recordings in Slices

Electrophysiological recordings were performed in brain slices from male and female CBA mice (P14-P21) as previously described (Olsen et al., 2018). The extracellular recording solution containing 125 mM NaCl, 2.5 mM KCl, 10 mM glucose, 1.2 mM NaH_2_PO_4_, 2 mM Na-pyruvate, 3 mM myo-inositol, 0.5 mM ascorbic acid, 26 mM NaHCO_3_, 2 mM CaCl_2_ and 1 mM MgCl_2_ was maintained at room temperature (22–25°C). Whole-cell recordings from DCN fusiform cells were obtained using 4–6 MΩ electrodes filled with an intracellular medium containing 116 mM K-gluconate, 0.2 mM EGTA, 40 mM HEPES, 5 Na_2_ phosphocreatine, 1 mM L-arginine, 1 mM MgCl_2_ and 0.1 mM CaCl_2_. Loose cell-attached recordings were obtained from fusiform cells in the voltage-clamp configuration using 4–6 MΩ electrodes filled with extracellular medium. The seal resistance was maintained at 20–60 MΩ and the baseline current was held at 0 pA by manually adjusting the holding voltage throughout a recording (Perkins, 2006). Extracellular field potentials were recorded in the current clamp configuration using 300–700 kΩ electrodes filled with extracellular medium. Fusiform cells were identified by their morphology, position and orientation within the fusiform layer (Zhang and Oertel, 1994). For whole-cell recordings, the passive properties of fusiform cells were additionally used for their identification (Pilati et al., 2012). Excitatory (EPSP) and inhibitory (IPSP) post-synaptic potentials were recorded as positive or negative voltage deflections, respectively from a holding potential of −65 mV (theoretical reversal potential of Cl^–^ using the aforementioned extra- and intra-cellular media was −105 mV at 22°C). Ongoing recordings of 5 min were band-pass filtered (2–100 Hz, Butterworth filter) and spontaneous post-synaptic potentials were detected by visually adjusting amplitude thresholds (positive and negative thresholds with respect to baseline for EPSPs and IPSPs, respectively). The detections were revised to ensure that all detections had the typical shape of subthreshold synaptic potentials, and that single EPSPs or IPSPs were not overlapping with neighboring EPSPs or IPSPs.

### Recording of Kv3 K^+^ Currents From Stably Transfected HEK Cells

Recordings of K^+^ currents were obtained from HEK 293 cells stably expressing hKv3.1b (NM 001112741.1) or hKv3.3 (NM 004977.2). Cells were grown on coverslips maintained in minimum essential medium (Sigma) containing 10% fetal bovine serum, 2 mM L-glutamine, 10 ml/l penicillin-streptomycin and 0.6 mg/ml geneticin (Gibco), maintained in a 5% CO_2_ incubator at 37°C. Cells were grown for 18–24 h before being transferred to an extracellular recording solution containing: 137 mM NaCl, 10 mM HEPES, 10 mM glucose, 1.8 mM CaCl_2_, and 1 mM MgCl_2_, with pH brought to 7.4 with 1 M NaOH. Electrophysiological recordings were obtained using a MultiClamp 700B amplifier (Molecular Devices) and digitized using a Digidata 1440A A/D convertor (Molecular Devices). Data were sampled at 50 kHz and low-pass filtered at 12 kHz. Whole-cell voltage clamp recordings were obtained using recording pipettes of 2–5 MΩ diameter and filled with intracellular medium containing: 120 mM KCl, 10 mM EGTA, 10 mM HEPES, 1.75 mM MgCl_2_, 5.37 mM CaCl_2_, and 4 mM Mg_2_ATP, with pH brought to 7.15–7.25 with 1 M KOH and final osmolarity brought to 290–300 mOsmol with sucrose. Series resistance was ∼10–15 MΩ and compensated by 70%. Conductance curves were created by plotting normalized conductance (G/G_*max*_) as a function of membrane potential (mV) and were fitted with a Boltzmann function (*B*) of the form:

$$B\left(V\right)=a+\frac{b-a}{1+exp^{\left(V_{50}-V\right)/c}}$$

where *a* and *b* are the minimal and maximal values of the normalized conductance (G/G_*max*_) respectively, *V*_50_ is the half-activation voltage, *V* is the membrane potential, and *c* is the slope factor. Conductance was obtained using a theoretical reversal potential of −85 mV for K^+^ currents.

### Pharmacology

Tetraethylammonium (TEA) chloride (Sigma-Aldrich) was used at a concentration of 0.5 mM to partially block Kv3 K^+^ currents (Erisir et al., 1999; Hernández-Pineda et al., 1999; Rosato-Siri et al., 2015), in addition to Kv1, Kv7, and BK Ca^2+^ currents (Brew and Forsythe, 1995; Johnston et al., 2010). AUT1 (Autifony Therapeutics Ltd.) was used to positively modulate Kv3 K^+^ currents (Rosato-Siri et al., 2015; Taskin et al., 2015; Brown et al., 2016; Chambers et al., 2017). AUT1 was dissolved in dimethylsulfoxide (DMSO) as 20 mM stock solutions (stored at −20°C) and subsequently diluted in ACSF on the day of an experiment, with DMSO concentrations matched for all recording solutions. AUT1 was used at a concentration of 10 and 30 μM when recordings were performed in HEK 293 cell cultures and brain slices, respectively. Higher concentrations of AUT1 were used for brain slice experiments to counteract the increased time required for the drug to diffuse into the relatively thick layers of tissue used in this study. These concentrations are in accordance with previous studies using recordings from brain slices and cell cultures (Rosato-Siri et al., 2015, EC50 of AUT1 on Kv3.1b = 5.33 μM; Brown et al., 2016). Kainic acid (750 nM) was used in some experiments to increase excitability (Cunningham et al., 2003). 1 mM kynurenic acid (Sigma-Aldrich) and 10 μM NBQX (2,3-dihydroxy-6-nitro-7-sulfamoylbenzo[f]quinoxaline-2,3-dione) (Ascent Scientific) were used to block excitatory synaptic transmission. Gabazine (Abcam) and strychnine (Abcam) were both used at a concentration of 10 μM to block inhibitory synaptic transmission. Tetrodotoxin (TTX) (Abcam) was used at concentration of 1 μM to block sodium currents and prevent action potential driven activity.

### Wavelet-Based Filtering and Generation of Wavelet-Filtered Current Stimuli

Spontaneous voltage fluctuations recorded from brain slices using the current clamp configuration were band-pass filtered using a Morlet wavelet (MW) convolution. The use of wavelets avoided ripple artifacts associated with plateau-shaped filters and helped retain the temporal resolution of the original signal (Mallat, 2008). MWs were constructed by multiplying a sine wave (carrier) with a Gaussian function (envelope):

$$MW\left(t\right)=a*sin\left(2\pi ft\right)*exp\left(\frac{-{\left(t-\mu \right)}^{2}}{2\sigma^{2}}\right)$$

where *t* is time, a*sin(2π*ft*) is a sine wave with *f* and *a* determining the center frequency and amplitude of the wavelet, respectively, and where $exp\left(\frac{-{\left(t-\mu \right)}^{2}}{2\sigma^{2}}\right)$ is a Gaussian function with μ and σ denoting the mean and standard deviation, respectively. σ was adjusted to produce wavelets with a bandwidth of 0.75 Hz in the frequency domain. Recordings were filtered and then divided into 30 s epochs. The peak-to-peak amplitude was determined for each frequency band within each epoch by taking the 6^∗^SD of the wavelet-filtered signal. The choice of MWs rather than a time resolved FFT kept consistency with the filters used for the stimulus generation in this study. Wavelet-filtered noise current stimuli were generated by the convolution of a MW with a discrete-time Ornstein-Uhlenbeck process (*X*) of the form:

$$X_{n+1}=\lambda_{1}X_{n}+\lambda_{2}\xi_{n+1}$$

where λ_1_ controls the degree of autocorrelation, and where ξ is a Gaussian white noise realization with amplitude controlled by λ_2_ (Uhlenbeck and Ornstein, 1930; Renshaw, 1987). To quantify the strength between action potential firing and the phase of the wavelet-filtered current stimuli, a measure of coherence was used as described in Fries et al. (1997). Using wavelet-filtered noise stimuli aimed at testing effects of noise frequency independently of noise amplitude. Therefore, wavelet-filtered noise stimuli were injected at a variety of peak-to-peak current amplitudes at each tested frequency and analysis was restricted to cases where the resulting peak-to-peak subthreshold voltage deflections in the cell were similar across stimulus frequencies. Subthreshold voltage deflections in response the wavelet stimuli used in this study (Figure 2D) were recorded as: 3 Hz: 10.8 ± 0.8 mV; 6 Hz: 10.4 ± 0.6 mV; 10 Hz: 11.1 ± 0.7 mV; 20 Hz: 10.1 ± 0.7 mV; and 40 Hz: 10.6 ± 0.8 mV.

### Local Field Potential Spike Detection

Extracellular field potentials were filtered using a second order elliptical filter (0.3–3 kHz) and action potentials were detected using Matlab’s “findpeaks” function, with the threshold (*Thr*) determined according to:

Thr=kH

where *k* was set to 5 and *H* is the median absolute deviation given by:

$$H=median\left(\frac{\left|fV\right|}{0.6745}\right)$$

where *fV* is the band-pass filtered signal (Rey et al., 2015). In cases where visual inspection of the data showed missed action potentials when performing peak detection, *k* was adjusted to 4.

### Quantification of Action Potential Synchrony

Spontaneous action potentials were recorded from a fusiform cell using the cell-attached recording configuration and from its neighboring cells in the fusiform layer using the extracellular recording configuration. Recordings were converted to time stamps and extracellular time stamps occurring within ±200 μs of a cell-attached time stamp were deleted, thus avoiding contaminating the extracellular with the cell-attached time stamps. Synchrony was quantified using the jitter-based synchrony index (JBSI), a method which has been shown to be independent of mean firing frequency, firing rate differentials between measured neurons and co-modulation of firing rates between neurons (Agmon, 2012). A synchrony window with span *W*_*s*_ (synchrony window) was first centered on each extracellular (target) spike (time stamp) and a value of 1 was assigned to a cell-attached (reference) spike if it fell within one or more synchrony windows. A synchrony value (*Nc*) was obtained by summing all values of 1. A jitter window with span *W*_*j*_ was then centered on each cell-attached (reference) spike. The jitter window was twice the size of the synchrony window span. For each jitter window, an area of overlap between jitter and synchrony windows was found and expressed as a probability. A measure of jitter (*J*) was obtained by summing all the jitter window probabilities. Finally, a normalized measure of synchrony (JBSI) is obtained using the equation:

$$JBSI=\beta \frac{N_{c}-J}{n}$$

where β is the ratio between *W*_*j*_ and *W*_*s*_ (i.e., 2), and n is the number of cell-attached (reference) spikes. Using this method, synchrony is restricted between 1 and −1 for maximal and minimal possible synchrony, respectively. For each recording, a measure of synchrony was obtained for synchrony spans ranging from 1 to 130 ms, thus resulting in a JBSI versus synchrony span curve. Synchrony was quantified using the maximum point of the JBSI curve and the time (1–130 ms) at the maximum point of the JBSI curve.

The JBSI was also used to assess synchrony between simulated spike times, where the temporal relationship between cell-attached and extracellular time stamps is precisely controlled. The goal of these simulations was to find the pattern of cell-attached and extracellular spike times that could replicate experimental observations (Figures 8E–G). Cell-attached time stamps were created using an evenly spaced vector of length 300 and occurring every 100 ms, thus representing a spike train consisting of 300 spikes and firing at a frequency of 10 Hz, a firing rate closely matching experimental conditions (Figure 8A, black, 9.0 ± 3.3 Hz). Synchrony was modified by adjusting the relative amount of extracellular time stamps occurring within and outside a 10 ms window surrounding each cell-attached time stamp.

### Imaging of Pre-synaptic Calcium Influx

Imaging of pre-synaptic calcium influx at the molecular-fusiform layer border was performed using slices prepared from male and female SyG36 mice which express SyGCaMP2-mCherry under a Thy1-2 promoter. SyG36 mice were bred on a C57/Blk6 background such that positive SyG36 heterozygote mice were crossed with WT, leading to litters containing approximately 50% of positive SyG36 mice (characterized by genotyping) (Al-Osta et al., 2018). Changes in SyGCaMP2 fluorescence (F) were triggered by placing a concentric bipolar stimulating electrode (outer/inner pole diameter: 125/25 μm, FHC, Inc., Bowdoin, ME, United States) under the control of a constant current stimulator (DS3, Digitimer, Hertfordshire, United Kingdom) in the molecular layer. An epifluorescence microscope was used to capture ensemble changes in calcium from pre-synaptic terminals, with GCaMP2 activated at a wavelength of 470 ± 20 nm using a custom-made LED, and emission collected at 520 ± 20 nm using an optiMOS Prime camera (Photometrics, Arizona, United States). The stimulation consisted of a 50 Hz pulse train of 2 s duration. A time series of images was collected every 100 ms within a 1200 by 450 pixel recording window (396.0 by 148.5 μm, respectively). Ensemble changes in calcium fluorescence captured using the epifluorescence microscope was performed without blockers of synaptic transmission. Fluorescence under these conditions therefore represents calcium influx occurring as a direct result of the stimulation and in response to indirect, polysynaptic excitation. Fluorescence was quantified as a function of stimulation intensity (Figures 4B,E), where the minimum and maximum intensity was adjusted individually for each brain slice to capture the initiation and plateau of the peak evoked fluorescence respectively. Peak evoked fluorescence versus stimulus intensity functions were fitted using a four-parameter Hill function of the form:

$$y=y_{0}+\frac{ax^{b}}{c^{b}+x^{b}}$$

where *y*_0_ is the intercept, *a* is the maximum, *c* is the half activation point (I_50_), *x* is the stimulus intensity and *b* is the Hill coefficient. All experimental data was adequately fit using the four-parameter Hill function (*r*^2^ > 0.85) thus ensuring that differences between experimental groups did not arise due to differences in the quality of fitting.

Measurements from individual pre-synaptic boutons were obtained using an upright multiphoton microscope equipped with a 20 × 1.0 NA objective (Zeiss MP7, Carl Zeiss Microscopy, GmbH, Jena, Germany), with GCaMP2 activated at an excitation wavelength of 920 nm and the emission collected at 520 ± 20 nm. Fluorescence was evoked in response to a 50 Hz, 2 s stimulus using a stimulation electrode placed in the molecular layer. A single stimulus intensity was chosen individually for each brain slice until a clear response could be detected, and subsequently maintained across drug conditions. The SARFIA toolbox based in IGOR software was used for the detection of individual fluorescence puncta (Dorostkar et al., 2010). Prior to puncta detection, images were background subtracted (taking a non-responsive portion of the image as the background), corrected for small movements using IGORs registration function, and filtered in the x-, y-, and time-axis domain using a median filter (3 × 3 × 3 pixels). Imaging of individual puncta was performed in presence of blockers of synaptic transmission (1 mM kynurenic acid, 10 μM NBQX, 10 μM strychnine and 10 μM gabazine). This enabled the recording of responses occurring as a direct result of the stimulation rather than of a polysynaptic excitation. Following SARFIA-based puncta detection, puncta which showed a significant increase in stimulus-evoked fluorescence using a one-sided Wilcoxon rank-sum test at *P* = 0.05 were considered as “responding” puncta, whereas all other puncta were considered as “non-responding.” To the confirm the effectiveness of this method, puncta were split according to whether maximum evoked fluorescence was above (responding) or below (non-responding) 4^∗^SDs above the baseline fluorescence, thus preventing puncta with large fluorescence peaks due to high baseline variability from becoming “responding” boutons.

Due to slight changes in focus and position of the brain slice over the duration of a recording, it was impracticable to record from the same boutons over long durations of time (and thus across different drug conditions). Therefore, in order to compare bouton properties across drug conditions in the same brain slice, bouton responses were captured in response to three stimulations (therefore obtaining three image stacks) for each drug condition. Small changes in z-focus were applied before each stimulation to maintain a set of anatomical landmarks in focus while also trying to capture as many bouton responses as possible within the immediate vicinity (*z*-axis). Histograms and cumulative distribution plots showing bouton response properties (Figures 4N–P) contain data from all three image stacks (three stimulations) in each drug condition and brain slice. The number of boutons for a single drug condition and brain slice (Figure 4Q) was taken from the image stack (of three total) with the greatest number of boutons.

For both imaging techniques (epifluorescence and multiphoton), the effects of photobleaching were compensated by subtracting a double exponential fitted to the fluorescence response that was absent of evoked fluorescence (pre- and post-stimulation). Fluorescence is presented as F/F_0_, where F_0_ is the average pre-stimulation fluorescence before bleach subtraction.

### Modeling the Effects of Increased Kv3 Conductance on Action Potential Firing Using a HH-Type Simulation

The effects of increased Kv3 K^+^ conductance on action potential firing was simulated using a Hodgkin and Huxley (HH) based model (Hodgkin and Huxley, 1952). The model comprised of a sodium current (I_*Na*_), a low-threshold potassium current (I_*K*_), a leak current (I_*L*_), and a Kv3 potassium current (I_*Kv*3_). Voltage (in mV) at any one time was modeled according to the equation:

$$V_{i+1}=V_{i}+\frac{di}{Cm}\left(-I_{Na\left(i+1\right)}-I_{K\left(i+1\right)}-I_{L\left(i+1\right)}-I_{Kv3\left(i+1\right)}\right)$$

where C_*m*_ is the membrane capacitance and the ionic currents are given by *I*_*Na*_ = *g*_*Na*_*m*^3^*h*(*V*−*V*_*Na*_), *I*_*K*_ = *g_*k*_n*^4^(*V*+*V*_*K*_), *I*_*L*_ = *g*_*L*_(*V*−*V*_*m*_−*V*_*L*_), and *I*_*Kv*3_ = *g*_*Kv*3_*n*^4^(*V*+*V*_*Kv*3_). *V*_*Na*_, *V*_*K*_, *V*_*L*_, and *V*_*Kv*3_ were taken as 50, −77, 10.6, and −81 mV, respectively.

The fast, high-voltage activating characteristics of Kv3 K^+^ channels were modeled according to Lien and Jonas (2003), where the alpha (α) rate constant for the Kv3 *n* gating variable was modeled using:

$$\alpha =a*\left(-\left(V+b\right)\right)/\left(\exp\left(-\frac{V+b}{c}\right)-1\right)$$

where *a* = 0.3 s^–1^, *b* = −4.18 mV^–1^, and *c* = 6.43 mV^–1^. Previous experimental observations have shown that an increase in Kv3 K^+^ conductance in presence of AUT1 (and partner compounds) results in an increase in action potential after-hyperpolarization while failing to affect any other action potential property, such as rise time, half-width or decay time (Chambers et al., 2017; Olsen et al., 2018). This process was effectively modeled with a beta (β) rate constant for the Kv3 *n* gating variable of the form:

$$\beta =d*\text{exp}\left(\frac{V}{80}\right)$$

where *d* = 0.011 s^–1^. This resulted in a Kv3 K^+^ conductance that activated late and fast during the rising phase of an action potential and subsequently remained active for ∼20 ms during the action potential after-hyperpolarization (Figure 7H), thus matching experimental observations (Olsen et al., 2018). Action potentials were triggered using a 50 Hz (100 pulses) pulse train, thus matching the stimulus used during calcium imaging. Stimulus intensity (μA⋅cm^–2^) was adjusted in order to produce action potentials in response to ∼95% of stimuli, with white noise added to the stimulus to produce variation in the times of action potential failure. The amplitude of the white noise was adjusted in order to produce a variation in stimulus pulse amplitude (in a pulse train) of ∼5% of their total amplitude (Figure 7I).

### Immunohistochemistry

Immunohistochemistry was performed on sagittal and coronal brainstem slices (14 μm) containing the DCN from SyG37 mice. Primary antibodies were anti-Kv3.1 mouse monoclonal (1:1000, Neuromab, Cat# 75-041), anti-Kv3.3 rabbit polyclonal (1:1000, Alomone, Cat# APC-102), SMI-312 mouse polyclonal (1:500, BioLegend, Cat# 837904), and anti-bassoon mouse monoclonal (1:250, Abcam, Cat# SAP7F407). Secondary antibodies consisted of goat anti-mouse Alexa 594 (1:1000, Invitrogen, Cat# R37121) and donkey anti-rabbit Alexa 488 (1:1000, Invitrogen, Cat# R37118). Brain slices were examined using an Olympus FV1000 confocal microscope. Alexa 488 was excited at a wavelength of 488 nm and the emission was collected between 500 and 545 nm. Alexa 594 was excited at a wavelength of 559 nm and the emission was collected between 575 and 620 nm. Spectral overlap between red and green channels was assessed by imaging brain slices containing only Alexa 488 or Alexa 594 (and their corresponding primary antibodies), using emission filters of 575–620 or 500–545 nm, respectively. The parallel collection of green (500–545 nm) and red (575–620 nm) fluorescence was avoided to further minimize confounding results due to spectral overlap. Slices in which the primary antibodies were omitted served as controls. Image analysis was performed using imageJ (Schneider et al., 2012) and FIJI (Schindelin et al., 2012) software. All images were converted to 8-bit format, convolved with a theoretical point spread function and thus performing image restoration by deconvolution (Landmann and Marbet, 2004), and background subtracted (using a section of an image containing no fluorescence as background). Co-localization between two fluorophores was quantified using Mander’s co-localization coefficients (Manders et al., 1993), where the Mander’s G coefficient is the percentage of pixels with green fluorescence that also contains red fluorescence and the Mander’s R coefficient is the percentage of pixels with red fluorescence that also contains green fluorescence. In instances of punctate fluorescence (Figures 6D,E), object-based co-localization was additionally performed after first transforming images to binary format using FIJI (using the “Make Binary” and “Fill holes” commands). Next, images were filtered using a median filter (2 × 2 pixels) and processed using a watershed segmentation algorithm to transform larger and very uncommon objects into smaller objects (Fiji’s “watershed” function). To assess whether the watershed segmentation procedure significantly affected results (i.e., whether splitting the larger, more uncommon objects into smaller objects altered the overall outcome of object-based co-localization), images in which watershed segmentation was omitted from the image processing stage were also analyzed. Object-based co-localization results (presented in Figure 6I) were not significantly different (*P* = 0.8) when watershed segmentation was omitted. The efficacy of this entire image processing procedure was confirmed by visual inspection. The percentage of objects in one image overlapping with objects in a second image (Objects co-localized (%)) was determined using

$$Objectscolocalized\left(\%\right)=\frac{\#objectscolocalized\left(1|2\right)}{Total\#objects\left(1\right)}*100$$

where “*# objects co-localized(1| 2)*” is a count of objects in image 1 which contains at least one pixel that overlaps with one or more objects in image 2, and “*Total # objects(1)*” is the total number objects in image 1.

### Statistics

Statistical analysis was performed using Graphpad Prism (Ver. 7), SPSS (Ver. 24) or Minitab (Ver. 20.3). All data were first tested for normality using a Shapiro–Wilk test. Paired *t*-tests (parametric) or Wilcoxon signed-rank tests (non-parametric) were used to test for differences between two paired data groups. A repeated measures ANOVA with Tukey *post hoc* tests (parametric) or a Friedman’s test with Dunn’s *post hoc* tests (non-parametric) were used to test for differences between three or more paired data groups. In a single case where one group contained a missing data point (Figure 2D), a linear mixed model was used as an alternative to the repeated measured ANOVA. A Mixed ANOVA was used when a dataset contained one within-subjects factor and one between-subjects factor (Figure 4Q). Greenhouse-Geisser correction of the degrees of freedom was performed for all ANOVAs where applicable (Greenhouse and Geisser, 1959). ANOVA results presented in the text are reported with this correction. A Pearson’s correlation coefficient test (parametric) or a Spearman’s rho test (non-parametric) were used to test the strength of a linear relationship between two data groups. For linear correlations, data from multiple recordings were frequently pooled and a single correlation was performed (Figures 2F,H). When a high variability existed between different recordings, individual correlations were performed for each recording separately and an average correlation coefficient was obtained using the method of Fisher’s z transformation (Fisher, 1992). Bonferroni-Holm correction of *P*-values was performed when multiple separate statistical tests were performed on the same data (Holm, 1979). For example, in Figures 3A–C, the spike-triggered average (STA) was compared between the control and TEA conditions using three separate paired *t*-tests on three different properties of the STA, and thus *P*-values from these *t*-tests were multiplied by 3. Where applicable, corrected *P*-values are presented in the text. An *F*-test for equal variances was performed to compare the variance between two groups. Minitab’s paired equivalence tests were used to test for equivalence of mean values between reference and test data. Data are presented as mean ± SD unless otherwise noted. N = number of animals. For electrophysiology, n = number of cells; for imaging and immunohistochemistry experiments, n = number of slices. F = number of images obtained from a single slice.

## Results

### Positive Modulation of Kv3 K^+^ Channels Inhibits Spontaneous and Evoked Synaptic Currents

**FIGURE 1:**
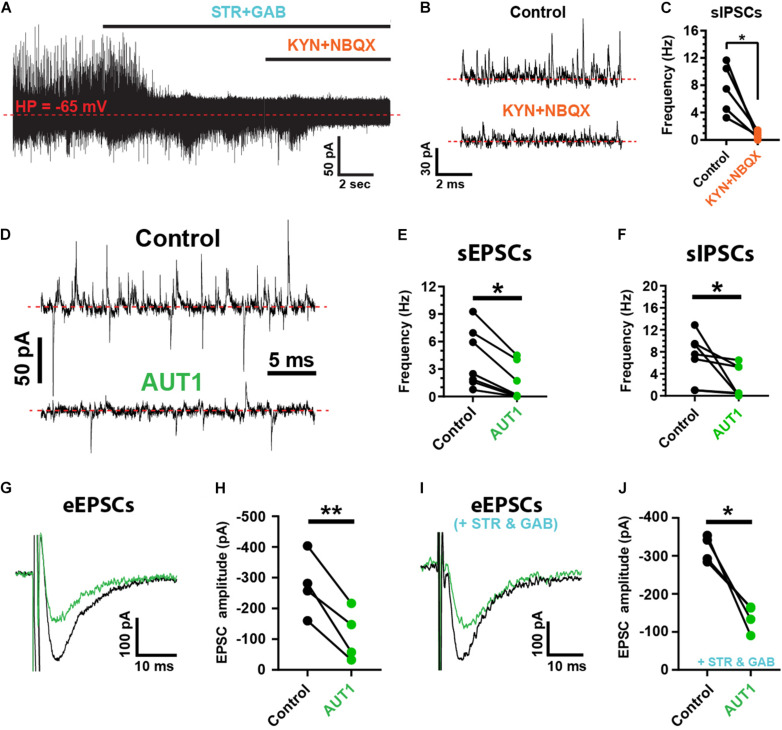
Positive modulation of Kv3 K^+^ currents reduces the frequency and amplitude of spontaneous and evoked synaptic currents. **(A)** Spontaneous inhibitory (sIPSCs, outward) and excitatory (sEPSCs, inward) post-synaptic currents recorded from fusiform cells held at –65 mV (red dashed line), using the whole-cell recording configuration. sISPCs were blocked by strychnine (STR) and gabazine (GAB), whereas sEPSCs were blocked by kynurenic acid (KYN) and NBQX. **(B)** Example recordings showing the suppression of sIPSCs following perfusion of blockers of excitatory synaptic transmission (KYN and NBQX). **(C)** Graph summarizing the inhibition of sIPSC frequency following kynurenic acid and NBQX perfusion (*n* = 5). **(D)** Spontaneous post-synaptic currents recorded from a fusiform cell in the control condition (top) and following perfusion of 30 μM AUT1 (bottom). **(E,F)** Presence of AUT1 reduced the frequency of sEPSCs (**E**, *n* = 6) and sIPSCs (**F**, *n* = 6). **(G,I)** Evoked excitatory post-synaptic currents (eEPSCs) recorded as inward currents from a fusiform cell in response to a single stimulus pulse (0.5 mA, 0.1 ms) delivered to the parallel fibers, in the control condition (black) and following perfusion of 30 μM AUT1 (green), in absence **(G)** and presence **(I)** of strychnine and gabazine. **(H,J)** Presence of AUT1 reduced the eEPSC peak amplitude in absence (**H**, *n* = 4) and in presence (**J**, *n* = 4) of strychnine and gabazine, respectively. Stimulus intensity ranged from 0.1 to 0.8 μA (0.1 ms) and was adjusted individually for each cell to produce a response ∼50% of the maximum. *p < 0.05, **p < 0.01.

Spontaneous and evoked synaptic currents were recorded from fusiform cells identified by their location in the DCN fusiform layer and by their characteristic response to current injection (Manis, 1990; Zhang and Oertel, 1994; Pilati et al., 2012). Fusiform cells were maintained at a holding potential of −65 mV. At that potential, outward spontaneous currents were blocked by strychnine (10 μM) and gabazine (10 μM), whereas inward spontaneous currents were blocked by kynurenic acid (1 mM) and NBQX (10 μM), identifying these currents as inhibitory and excitatory post-synaptic currents (IPSCs and EPSCs), respectively (Figure 1A). Spontaneous IPSC (sIPSC) frequency was decreased by kynurenic acid and NBQX (Figures 1B,C, control: 7.5 ± 3.6 Hz; kynurenic acid and NBQX: 0.7 ± 0.6 Hz, *n* = 5, *N* = 3, paired *t*-test: *t*(4) = 4.0, *P* = 0.016) demonstrating a di-synaptic origin of the sIPSCs similarly to IPSCs evoked by parallel fiber stimulation (Doiron et al., 2011; Tang and Trussell, 2017). We next tested the role of AUT1, a positive modulator of Kv3 K^+^ currents (Rosato-Siri et al., 2015; Brown et al., 2016). AUT1 (30 μM) reduced the frequency of spontaneous EPSCs (sEPSCs; Figures 1D,E, control: 4.1 ± 3.2 Hz; AUT1: 1.5 ± 2.0 Hz, *n* = 7, *N* = 5, Wilcoxon signed rank test: *W* = −28.0, *P* = 0.03) and spontaneous IPSCs (sIPSCs, Figures 1D,F, control: 6.8 ± 4.4 Hz to 2.6 ± 2.9 Hz, *n* = 7, *N* = 5, Wilcoxon signed rank test: *W* = −28.0, *P* = 0.03). AUT1 also reduced the amplitude of post-synaptic currents evoked by parallel fiber stimulation in the absence of synaptic blockers (Figures 1G,H, control: −275.5 ± 100.2 pA; AUT1: ^–^113.4 ± 84.6 pA, *n* = 4, *N* = 2, paired *t*-test: *t*(3) = 6, *P* = 0.009) and in the presence of strychnine and gabazine (Figures 1I,J, control: –318.0 ± 34.7 pA; AUT1: −137.9 ± 34.9 pA, *n* = 4, *N* = 2, paired *t*-test: *t*(3) = 5.3, *P* = 0.013), demonstrating its direct and indirect effect on parallel fiber mediated excitatory and inhibitory synaptic transmission, respectively.

### Positive Modulation of Kv3 K^+^ Channels Inhibits High Frequency Noise of Synaptic Origin

**FIGURE 2:**
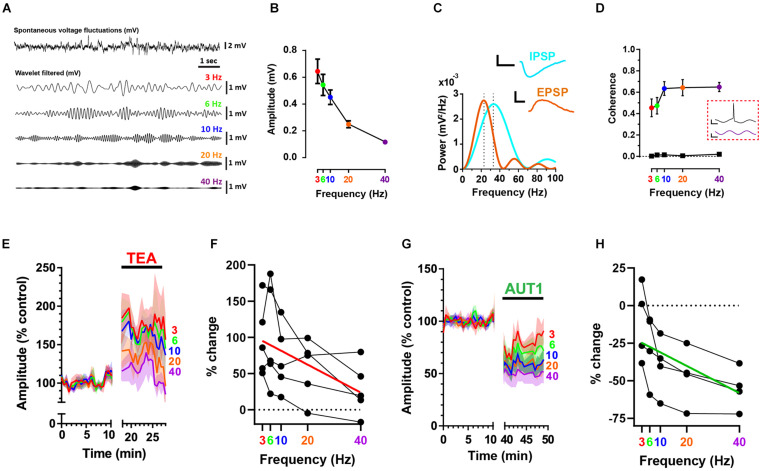
Positive modulation of Kv3 K^+^ currents reduces high frequency voltage fluctuations at the fusiform cell membrane. **(A)** Whole-cell current clamp recordings showing a portion of spontaneous voltage fluctuations (top trace) recorded from a fusiform cell filtered using cosine wavelets (lower traces) centered at 3 (red), 6 (green), 10 (blue), 20 (orange), and 40 Hz (purple). **(B)** Peak-to-peak wavelet-filtered voltage amplitude as a function of wavelet center frequency (*n* = 9). Error bars = SEM. **(C)** Power spectra (mV^2^/Hz) for the mean excitatory (EPSP, brown) and inhibitory (IPSP, cyan) spontaneous post-synaptic potentials (*n* = 9). Spontaneous EPSPs and IPSPs are represented as Gaussian functions with frequency domains centered at 23 and 33 Hz, respectively (illustrated by the vertical dashed lines). The mean sEPSP and sIPSP are shown in the inset. Horizontal scale bars: 10 ms; vertical scale bars: 1 mV. **(D)** Coherence between action potential firing and wavelet-filtered noise stimuli as a function of wavelet center frequency (*n* = 7). Bottom trace (black squares) depicts the coherence for identical spike trains but measured in relation to newly generated wavelet-filtered noise stimuli (i.e., not used in the experiments). Data show the mean (error bars = SD) from 100 wavelet stimuli at each frequency. Inset (red dashed box): action potential (top) elicited in response to a portion of wavelet-filtered noise centered at 40 Hz (bottom). Horizontal scale bars: 10 ms; upper and lower vertical scale bars: 20 mV and 300 pA, respectively. **(E,G)** Peak-to-peak subthreshold membrane voltage fluctuation amplitudes were recorded in the control condition and following the perfusion of 0.5 mM TEA **(E)** or 30 μM AUT1 **(G)**. Shaded error bars = SEM. **(F,H)** Percent change (relative to control) of subthreshold voltage fluctuation amplitudes as a function of frequency, for amplitudes measured in presence of TEA (**F**, *n* = 5) and AUT1 (**H**, *n* = 5). Red **(F)** and green **(H)** lines represent linear fits to the data. **(A–H)** Holding potential: –65 mV.

Spontaneous, subthreshold voltage fluctuations recorded from fusiform cells using the whole-cell current clamp configuration (Figure 2A, top) were band-pass filtered using a convolution with wavelets centered at 3, 6, 10, 20, and 40 Hz (Figure 2A, bottom) to allow measuring the effects of Kv3 K^+^ channel modulation on synaptic noise as a function of frequency. Figure 2B shows that voltage fluctuation amplitude was inversely related to frequency (*n* = 9, *N* = 5, RM ANOVA: *F*(1.2, 9.4) = 34.8, *P* < 1 × 10^–3^). Recordings were obtained using a holding potential of −65 mV, thus positive and negative voltage deflections represented excitatory (EPSP) and inhibitory (IPSP) post-synaptic potentials, respectively (Figure 1A). At this holding voltage, the mean EPSP and IPSP waveforms (isolated as described in the materials and methods) were represented in the frequency domain by Gaussian functions centered at 23 and 33 Hz, respectively (Figure 2C, *n* = 9, *N* = 4, power spectrum obtained from the average EPSP and IPSP across cells), thus suggesting that high frequency (>10 Hz) voltage fluctuations are predominantly composed of synaptic potentials. The relationship between the frequency of membrane potential fluctuations and action potential firing was next investigated using a measure of coherence (Fries et al., 1997; Higgs and Spain, 2011). Action potentials were triggered by white noise current injections that were band-pass filtered at the same frequencies observed in Figures 2A,B using wavelet-based filtering. Coherence between the wavelet-filtered noise stimuli and the firing of action potentials increased as a function of noise current injection frequency (Figure 2D, *n* = 7, *N* = 4, linear mixed model with repeated measures: *F*(1.7, 9.8) = 17.7, *P* < 1 × 10^–3^), indicating that synaptic noise composed of high (10–40 Hz) frequencies are more likely to trigger action potentials.

In accordance with our previous research showing an increase in synaptic bombardment following the non-selective blockade of K^+^ currents using 0.5 mM TEA (Olsen et al., 2018), TEA (0.5 mM) increased membrane voltage fluctuation amplitudes (Figure 2E). Despite wavelet center frequency failing to affect the amplitude of voltage fluctuations in presence of TEA (Figure 2F, *n* = 5, *N* = 2, RM ANOVA: *F*(1.5, 6.2) = 4.6, *P* = 0.07), TEA preferentially amplified the amplitude of membrane voltage composed of lower center frequencies (Figure 2F, red line, Pearson correlation coefficient *r* = −0.53, *P* = 0.0068), an effect which is surprising when considering that EPSPs and IPSPs recorded from fusiform cells are principally composed of frequencies exceeding 10 Hz (Figure 2C). This could be due to 0.5 mM TEA blocking additional low voltage (i.e., −50 mV) activated Kv1 K^+^ channels (Hopkins, 1998) present in fusiform cells (Rusznák et al., 2008). Increased blockade of Kv1 K^+^ channels would increase the membrane resistance and consequently the membrane time constant, thus shifting membrane frequencies toward lower frequencies.

In contrast, modulation of Kv3 channels using AUT1 decreased membrane voltage fluctuation amplitudes (Figure 2G), with a larger effect observed at high frequencies (Figure 2H, *n* = 4, *N* = 3, RM ANOVA: *F*(2.0, 5.9) = 21.2, *P* < 0.01; green line, Spearman rho: *r* = 0.64, *P* = 0.0025). In summary, Kv3 K^+^ current activation inhibits both excitatory and inhibitory synaptic transmission to fusiform cells, causing a preferential reduction of higher frequency (≥10 Hz) voltage fluctuations on the fusiform cell membrane (Figures 2G,H). Action potential coherence is strongest at these higher frequency voltage fluctuations (Figure 2D), thus demonstrating that pre-synaptic Kv3 K^+^ current modulation regulates the occurrence of synaptic noise at the post-synaptic site which is effective at triggering action potentials.

### The Role of Post-synaptic Kv3 K^+^ Current in Modulating the Strength Between Synaptic Noise and Action Potential Firing

**FIGURE 3:**
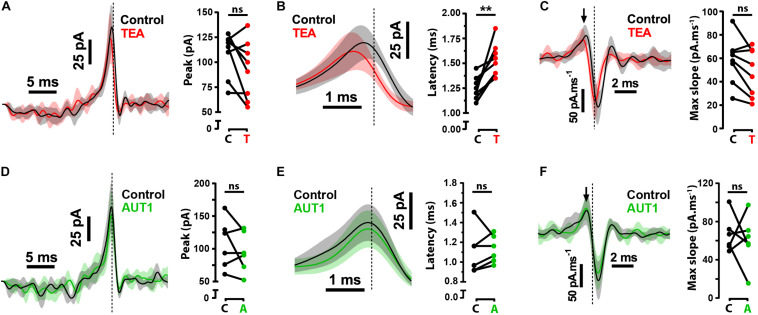
Effects of Kv3 K^+^ current modulation on the spike-triggered average (STA). **(A,D)** Left: Whole-cell current clamp recordings showing the average STA in the control condition (black) and following perfusion of TEA (**A**, red) or AUT1 (**D**, green). Right: Neither TEA **(A)** nor AUT1 **(D)** affected the peak STA. **(B,E)** Left: The STA depicted on an expanded time scale showing the latency between the STA peak and the time of action potential occurrence in control (black) and following TEA **(B)** or AUT1 perfusion **(E)**. Right: Perfusion of TEA **(B)** or AUT1 **(E)** increased or failed to affect the latency between the STA peak and the time of action potential occurrence respectively. **(C,F)** Left: The first derivative of the STA quantifying the peak (maximum) slope of the STA (black arrow), in the control and TEA conditions **(C)** and the control and AUT1 conditions **(F)**. Right: data plot showing the absence of effect of TEA **(C)** and AUT1 **(F)**. **(A–F)** Vertical dashed lines in STA plots indicate the time of action potential occurrence. Shaded error bars = SD. **(A,B,D,E)** STAs have been low pass filtered at 500 Hz for visualization. Peak and latency values are obtained from unfiltered STAs. **(A–C)**: *n* = 8; **(D–F)** *n* = 6. **p < 0.01.

Although our results demonstrate a modulatory effect of AUT1 on pre-synaptic Kv3 K^+^ channels, they do not exclude the possibility of an additional post-synaptic role on the initiation of fusiform cell action potentials. To determine the role of post-synaptic Kv3 K^+^ currents on the initiation of action potentials, we calculated the reverse correlation of the current waveform that caused the initiation of action potentials in response to band-pass filtered (0.1–1500 Hz) white noise current injections (1 nA peak-to-peak, 500 ms). The white noise current injection was combined with a DC pedestal from which the amplitude was chosen to maintain a firing frequency of ∼15 Hz across drug conditions. The reverse correlation, otherwise known as the spike-triggered average (STA), was measured in the control condition and following the perfusion of TEA or AUT1 (Figure 3). Perfusion of 0.5 mM TEA failed to affect the STA amplitude (Figure 3A, control: 109.0 ± 21.7 pA, TEA: 92.3 ± 29.3 pA, *n* = 8, *N* = 4, Wilcoxon signed rank test: *W* = −24.0, *P* = 0.33) and the STA rising slope (Figure 3C, control: 58.0 ± 19.6 pA.ms^–1^, TEA: 47.7 ± 30.3 pA.ms^–1^, *n* = 8, *N* = 4, paired *t*-test: *t*(7) = 2.6, *P* = 0.11). It did however increase the latency between the time of action potential occurrence and the STA peak (Figure 3B, control: 1.3 ± 0.1 ms, TEA: 1.5 ± 0.2 ms, *n* = 8, *N* = 4, paired *t*-test: *t*(7) = 4.6, *P* = 0.0075). Non-selective blockade of K^+^ channels therefore affects the initiation of action potential firing via post-synaptic mechanisms. In contrast, AUT1 failed to affect the STA peak (Figure 3D, control: 108.0 ± 37.6 pA, AUT1: 94.6 ± 30.8 pA, *n* = 6, *N* = 3, paired *t*-test: *t*(5) = 1.2, *P* = 0.9), the latency between the time of action potential occurrence and the STA peak (Figure 3E, control: 1.1 ± 0.2 ms, AUT1: 1.1 ± 0.14 ms, *n* = 6, *N* = 3, paired *t*-test: *t*(5) = 0.4, *P* = 2.1), or the STA rising slope (Figure 3F, control: 66.4 ± 18.8 pA.ms^–1^, AUT1: 60.0 ± 26.6 pA.ms^–1^, *n* = 6, *N* = 3, paired *t*-test: *t*(5) = 0.5, *P* = 2.0). Equivalence tests were additionally performed to confirm the lack of effect of AUT1 (Figures 3D–F, right). The mean STA peak in the AUT1 condition (94.6 ± 30.8 pA) fell within the 95% equivalence bounds for the mean STA peak for the control condition (lower bound: 72.0 pA; upper bound: 117.2 pA); the mean latency between the time of action potential occurrence and the STA peak for the AUT1 condition (1.1 ± 0.14 ms) fell within the 95% equivalence bounds for the mean latency between the time of action potential occurrence and the peak of the STA for the control condition (lower bound: 1.0 ms; upper bound: 1.2 ms) and the mean STA rising slope in the AUT1 condition (60.0 ± 26.6 pA.ms^–1^) fell within the 95% equivalence bounds for the mean STA rising slope in the control condition (lower bound: 32.1 pA.ms^–1^; upper bound: 88.0 pA.ms^–1^). Positive modulation of Kv3 K^+^ currents fails to affect the STA in response to white noise current injections, as expected considering the high voltage activated characteristics of Kv3 K^+^ channels (Rudy and McBain, 2001; Olsen et al., 2018). The lack of effect of AUT1 illustrates that the modulation of Kv3 K^+^ channels at the fusiform cell membrane fails to affect the initiation of action potentials at these cells as measured using the STA in response to white noise current injections. Moreover, this confirms that the actions of AUT1 on the initiation of fusiform cell action potentials arises from a pre-synaptic regulation of Kv3 K^+^ channels.

### Positive Modulation of Kv3 K^+^ Channels Decreases Ca^2+^ Fluorescence in Pre-synaptic Puncta Responding to Electrical Stimulation

**FIGURE 4:**
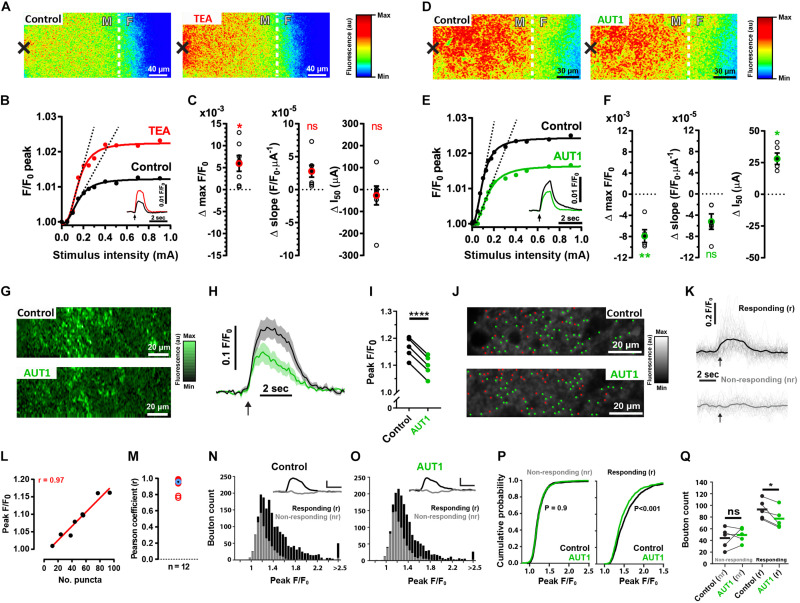
Effects of positive Kv3 K^+^ current modulation on the SyGCaMP2 fluorescence response following parallel fiber stimulation. **(A,D)** Heat maps depicting an increase in SyGCaMP2 fluorescence in response to parallel fiber stimulation (500 μA, 50 Hz, 100 pulses) in the control condition (**A,D**, left) and following perfusion of TEA (**A**, right) or AUT1 (**D**, right). The black crosses depict the position of the stimulation electrode. The white dashed lines show the border between the molecular (white “M”) and fusiform (white “F”) layer. **(B,E)** SyGCaMP2 fluorescence maximal peak response as a function of stimulation intensity (mA) for the recording shown in panels **(A,D)**, respectively. Peak responses are fitted using a four parameter Hill function. Dashed lines are linear regressions fitted to the linear, rising phase of the transfer function. Insets: SyGCaMP2 fluorescence in response to a 500 μA pulse train (50 Hz, 2 s) current intensity stimulation (time of delivery illustrated by black arrows), in the control condition (black traces) and following perfusion of TEA (**B**, red trace) or AUT (**E**, green trace). **(C,F)** Change in maximal F/F_0_ (obtained from the Hill function, left), the rising slope (obtained from the linear regression fits to the rising phase of the Hill function, middle) and the half activation point (obtained from the Hill function, right) following perfusion of TEA **(C)** or AUT1 **(F)**. Empty black circles represent results from a single brain slice and filled black circles with red **(C)** or green **(F)** outlines represent the mean (**C**, *n* = 7; **F**, *n* = 5). **(G)** Multiphoton microscopy images showing changes in calcium fluorescence within an area bordering the molecular and fusiform layers, following parallel fiber stimulation (750 μA, 50 Hz, 100 pulses) in the control (top) and AUT1 (bottom) conditions. **(H)** Average SyGCaMP2 fluorescence as a function of time, evoked in response to a 50 Hz (100 pulses) parallel fiber stimulation (time of delivery indicated of black arrow), in the control (black) and AUT1 (green) conditions (*n* = 5). **(I)** Perfusion of AUT1 decreased the peak fluorescence. **(J)** Multiphoton microscopy images (top: control; bottom: AUT1) showing a field between the molecular and fusiform layer, with green and red dots representing the location of responding and non-responding boutons, respectively. **(K)** Single bouton fluorescence as a function of time for the responding (top) and non-responding (bottom) boutons depicted in panel **(J)** (top). The thick lines represent the average responses. The black arrows denote the time at the start of the stimulation. **(L)** Peak fluorescence from the entire imaging window plotted against the number of responding boutons for a single brain slice. Individual points were obtained from current intensities ranging from 100 to 700 μA. The correlation coefficient between these two variables was 0.97. **(M)** Similarly obtained Pearson correlation coefficients (red circles) for 12 brain slices. The blue circle with the black center shows the average correlation coefficient (*r* = 0.96). **(N,O)** Histograms showing the distribution of peak values (peak F/F_0_) for responding (black) and non-responding (gray) boutons in the control **(N)** and AUT1 **(O)** conditions. Insets show the mean bouton fluorescence trace for the non-responding (gray) and responding (black) boutons. Horizontal and vertical scale bars are 2 s and 0.2 F/F0 respectively. **(P)** Cumulative probability plots showing the distribution of peak values (peak F/F0) for non-responding (left) and responding (right) boutons in the control (black) and AUT1 (green) conditions. The printed *p* values show results from Kolmogorov-Smirnov tests between the control and AUT1 conditions. **(Q)** Perfusion of AUT1 failed to affect the number of non-responding boutons (left) but did cause a significant decrease in the number of responding boutons (right) **(C,F)** Error bars = SEM. **(H)** Shaded error bars = SEM. *p < 0.05, **p < 0.01, ****p < 0.0001

We next characterized the pre-synaptic effects of Kv3 K^+^ current modulation using brain slices originating from SyG37 mice which express SyGCaMP2-mCherry, a genetically encoded ratiometric calcium indicator that exclusively targets pre-synaptic terminals via fusion to the vesicular protein synaptophysin (Al-Osta et al., 2018). Electrical stimulation administered to the molecular layer to activate parallel fibers increased SyGCaMP2 fluorescence at the fusiform-molecular layer border (Figures 4A,D left). Perfusion with 0.5 mM TEA increased the maximum amplitude of the fluorescence response compared to the control condition (Figure 4A right), an effect evident over a range of different stimulus intensities (Figures 4B,C left, control: 1.032 ± 0.030 F/F_0_; TEA: 1.038 ± 0.033 F/F_0_, *n* = 7, *N* = 4, Wilcoxon signed rank test: *W* = 28.0, *P* = 0.047; Two sample *F*-test for equal variances: *F*(6, 6) = 0.85, *P* = 0.85). TEA did not significantly affect the rising slope of a Hill function fitting the peak fluorescence response to increasing intensities of stimulation (Figures 4B,C middle, control: 5.9 × 10^–5^ ± 3.5 × 10^–5^ F/F_0_⋅μA^–1^; TEA: 8.4 × 10^–5^ ± 5.0 × 10^–5^ F/F_0_⋅μA^–1^, *n* = 7, *N* = 4, paired *t*-test: *t*(6) = 3.1, *P* = 0.06; Two sample *F*-test for equal variances: *F*(6, 6) = 0.50, *P* = 0.41; rising slope illustrated using the dashed lines in panel B; all linear fits had a goodness of fit *r*^2^ value > 0.95), nor did TEA affect the half activation point of the Hill function (Figures 4B,C right, control: 370.7 ± 294.0 μA; TEA 344.1 ± 266.9 μA, *n* = 7, *N* = 4, paired *t*-test: *t*(6) = 0.62, *P* = 1.7; Two sample *F*-test for equal variances: *F*(6, 6) = 1.2, *P* = 0.82). In contrast, perfusion with 30 μM AUT1 decreased the peak fluorescence over the entire range of stimulus intensities (Figure 4D, right). AUT1 significantly decreased the maximum evoked fluorescence (Figures 4E,F left, control: 1.026 ± 0.014 F/F_0_; AUT1 1.018 ± 0.012 F/F_0_, *n* = 5, *N* = 3, paired *t*-test: *t*(4) = 6.4, *P* = 0.009; Two sample *F*-test for equal variances: *F*(4, 4) = 1.4, *P* = 0.78). The initial slope of the Hill function was not significantly affected by the presence of AUT1 (Figures 4E,F middle, control: 14.6 × 10^–5^ ± 10.1 × 10^–5^ F/F_0_⋅μA^–1^; AUT1: 9.4 × 10^–5^ ± 7.0 × 10^–5^ F/F_0_⋅μA^–1^, *n* = 5, *N* = 3, paired *t*-test: *t*(4) = 3.6, *P* = 0.07; Two sample *F*-test for equal variances: *F*(4, 4) = 2.1, *P* = 0.50; rising slope illustrated using the dashed lines in panel E. All linear fits had a goodness of fit *r*^2^ value > 0.95), whereas the half activation point of the Hill function increased in presence of AUT1 (Figures 4E,F right, control: 109.9 ± 22.0 μA; AUT1: 138.0 ± 27.4 μA, *n* = 5, *N* = 5, paired *t*-test: *t*(4) = 6.2, *P* = 0.01; Two sample *F*-test for equal variances: *F*(4, 4) = 0.65, *P* = 0.68).

The results presented so far were obtained using an epifluorescence microscope and therefore represent the ensemble response from thousands of pre-synaptic boutons within a relatively thick layer of tissue. We next measured SyGCaMP2 responses using a multiphoton microscope (lens NA = 1; lens magnification = ×20) where the increased magnification and improved optical sectioning limits the contribution of out of focus light sources and thus allows responses from individual boutons to be measured (Al-Osta et al., 2018). Recordings were additionally obtained in presence of blockers of synaptic transmission, thus enabling the recording of SyGCaMP2 responses occurring as a direct result of the stimulation in absence of polysynaptic excitation. Measurements taken from the whole field of view representing an area of 212.5 by 26.6 μm (Figure 4G) produced responses that were entirely consistent with the ensemble results described above. At a fixed stimulation intensity, AUT1 produced a 43% (±12%) reduction in the peak fluorescence response compared to the control condition (Figures 4H,I, control: 1.17 ± 0.04 F/F_0_; AUT1: 1.10 ± 0.04 F/F_0_, *n* = 5, *N* = 3, paired *t*-test: *t*(4) = 50.4, *P* < 1 × 10^–3^). This large reduction indicates a powerful modulatory effect of AUT1 on pre-synaptic calcium which will reduce levels of synaptic transmission (Heidelberger et al., 1994; Neher and Sakaba, 2008), and subsequently the excitability of post-synaptic neurons in this circuit (Figures 1, 2).

We next sought to determine the role of AUT1 on individual boutons. Using a thresholding method previously described (Dorostkar et al., 2010; Al-Osta et al., 2018; Pereda et al., 2019), punctate regions of fluorescence (puncta) representing putative boutons were identified and subsequently grouped into responding and non-responding boutons (see section “Materials and Methods”). Figure 4J shows images of the molecular layer in the control (top) and AUT1 (bottom) conditions, with green dots representing responding boutons and red dots representing non-responding boutons. Figure 4K shows example bouton traces for responding (top) and non-responding (bottom) boutons in the control condition. We sought to determine if the number of responding boutons obtained using our separation technique was consistent with the size of the fluorescence response from the entire imaging window following stimulation (Figures 4H,I). The number of responding boutons was compared to the average fluorescence from the entire imaging window in response to a series of increasing current stimulations. A linear relationship was observed between the size of the ensemble response and the number of responding boutons (Figure 4L). The average correlation coefficient (r) for 12 brain slices was 0.93 ± 0.08 (Figure 4M). These results strongly suggest that fluorescence captured from the entire imaging window following stimulation of the molecular layer reflects the number of responding boutons. The next step consisted in measuring the effect of AUT1 on the number of responding and non-responding boutons. For each brain slice, bouton peak values were obtained from three image stacks (and therefore three stimulations) in the control and AUT1 conditions (see section “Materials and Methods”). Histograms in Figures 4N,O represent all bouton peak values for the control (Figure 4N) and AUT1 (Figure 4O) conditions, where the black bars represent a count of responding boutons and the gray bars represent a count of non-responding boutons. Plotting the peak F/F_0_ for all non-responding boutons in a cumulative distribution revealed almost identical distributions for the control and AUT1 conditions (Figure 4P, left). Testing for a difference in distributions using a Kolmogorov–Smirnov test revealed a non-significant effect (control: *n* = 728 boutons, AUT1: *n* = 872 boutons, *P* = 0.92), thus reflecting no change in non-responding bouton peak amplitude in the AUT1 condition. In contrast, cumulative distribution plots for responding boutons (Figure 4P, right) revealed a negative, leftward shift in peak amplitude (F/F_0_) in the AUT1 condition. This result was paired with a significant difference in distributions using a Kolmogorov–Smirnov test (control: *n* = 1190 boutons, AUT1: *n* = 984, *P* < 1 × 10^–3^), and thus reflected a decrease in responding bouton peak amplitude for the AUT1 condition. To quantify the change in the number of non-responding and responding boutons following the perfusion of AUT1, the number of non-responding and responding boutons for each brain slice was obtained for each drug condition from the image stack with the maximum number of responding boutons (see section “Materials and Methods”). A mixed ANOVA with drug condition (control or AUT1) as the within-subjects factor, and response type (non-responding or responding) as the between-subjects factor revealed a significant interaction (*F*(1, 8) = 6.2, *P* = 0.037). Subsequent *post hoc* tests revealed a significant decrease in the number of responding boutons in the presence of AUT1 (Figure 4Q, right, control: 93.0 ± 16.5; AUT1: 77.2 ± 17.4, paired *t*-test: *t*(4) = 3.5, *P* = 0.048). In contrast, the number of non-responding boutons did not change following the perfusion of AUT1 (Figure 4Q, left, control: 45.2 ± 18.0; AUT1: 50.6 ± 12.4, left, *n* = 5, *N* = 3, paired *t*-test: *t*(4) = 0.75, *P* = 0.98). Similar results were also obtained when responding and non-responding boutons were split according to whether a bouton’s maximum evoked fluorescence occurred above or below 4^∗^SDs of the baseline respectively (mixed ANOVA, drug condition × response type interaction, *F*(1, 8) = 83.5, *P* < 1 × 10^–3^). AUT1 caused a significant decrease in responding boutons (control: 72.8 ± 20.5; AUT1: 50.0 ± 18.6, *n* = 5, *N* = 3, paired *t*-test: *t*(4) = 12.67, *P* < 1 × 10^–3^), but did not affect non-responding boutons (control: 65.6 ± 18.0; AUT1: 77.8 ± 12.3, *n* = 5, *N* = 3, Wilcoxon signed-rank test: *W* = 15.0, *P* = 0.13).

Absolute baseline fluorescence was measured and found to be unchanged following the perfusion of AUT1 (control: 48.2 ± 16.0 F; AUT1: 45.3 ± 16.0 F, *n* = 5, *N* = 3, paired *t*-test: *t*(4) = 1.12, *P* = 0.32), suggesting that bleaching over the time course of the experiment was minimal and therefore unlikely to have contributed significantly to the effects observed.

From these experiments, we conclude that potentiation of Kv3 K^+^ currents using AUT1 reduces the number of active pre-synaptic boutons in response to stimulation of the parallel fibers.

### A Proportion of Pre-synaptic Boutons in the Molecular Layer Contain Kv3.3 K^+^ Channels

We hypothesized that the reduction in the number of responding boutons in response to AUT1 was the result of Kv3 K^+^ channels present on the parallel fiber pre-synaptic boutons and/or axons. We therefore used immunolabeling against two subclasses of Kv3 K^+^ channels (Kv3.1b and Kv3.3) located in the auditory brainstem (Rudy and McBain, 2001; Zettel et al., 2007; Desai et al., 2008; Choudhury et al., 2020) to test this possibility.

**FIGURE 5:**
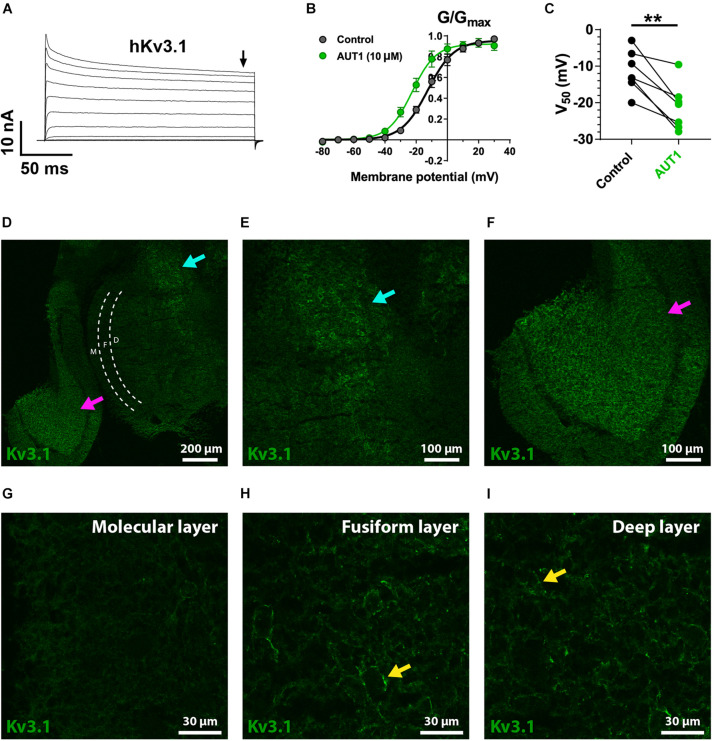
Kv3.1 K^+^ channel functional characteristics and expression in the dorsal cochlear nucleus. **(A)** Whole-cell voltage clamp recordings showing outward currents recorded form HEK 293 cells stably expressing Kv3.1b K^+^ channels (hKv3.1) in response to command voltages stepped from –80 to 40 mV in 10 mV increments. **(B)** Normalized conductance (G/G_*max*_) curves plotted as a function of the step potentials (mean ± SEM), in the control condition (black) and following perfusion of 10 μM AUT1 (green). Currents were measured at the end of the voltage step (arrow in panel **A**). Data were fitted using a Boltzmann function. **(C)** Half-activation voltages (V_50_) obtained from the Boltzmann curves in the control (black) and AUT1 (green) conditions. Perfusion of AUT1 caused a significant shift in V_50_ to more negative voltages (*n* = 7). **(D)** Confocal image from a sagittal brainstem slice (12 μm) showing weak labeling of Kv3.1b K^+^ channels in the DCN. The DCN is partitioned into its three layers (molecular layer: white “M”; fusiform layer: white “F”; deep layer: white “D”) using white dashed lines. The cyan and magenta arrows indicate labeling of Kv3.1b K^+^ channels in the brainstem at a location medial with respect to the DCN (cyan arrow) and in the cerebellar granule cell layer (magenta arrow). **(E,F)** Panel **(D)** with increased zoom showing locations marked by the cyan (**E**, top right in panel **D**) and magenta (**F**, bottom left in panel **D**) arrows. **(G–I)** Images of increased magnification showing the molecular **(G)**, fusiform **(H)**, and deep **(I)** layers. The yellow arrows **(H,I)** reveal a weak expression of Kv3.1b K^+^ channels on cell bodies. **p < 0.01.

**FIGURE 6:**
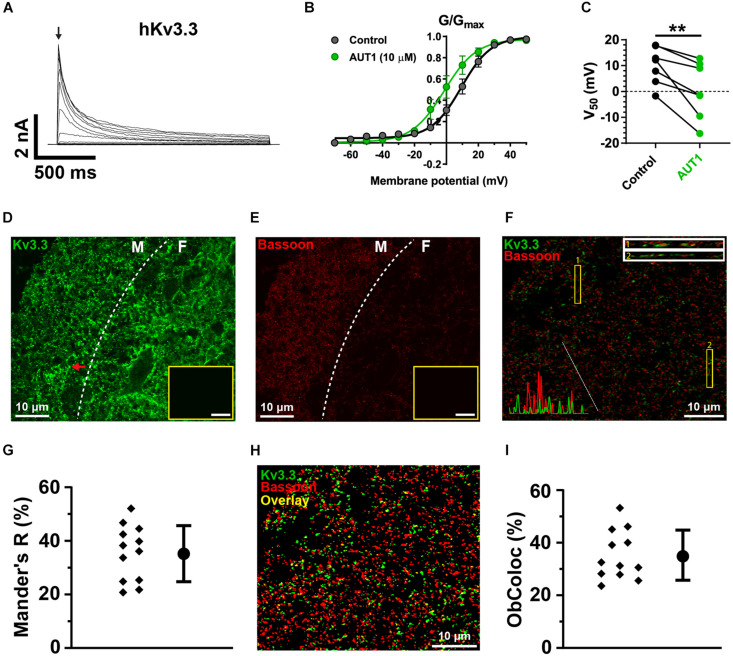
Kv3.3 K^+^ channels are present in a third of parallel fiber pre-synaptic terminals. **(A)** Whole-cell voltage clamp recordings showing outward currents recorded from HEK 293 cells stably expressing Kv3.3 K^+^ channels (hKv3.3) in response to voltage steps from –70 to 50 mV in 10 mV increments. **(B)** Normalized conductance (G/G_*max*_) curves plotted as a function of the step potentials (mean ± SEM) in the control (black) and AUT1 (green) conditions. Currents were measured at the initial peak of the outward currents (arrow in panel **A**). Data were fitted using a Boltzmann function. **(C)** AUT1 caused a significant shift in V_50_ to more negative voltages (*n* = 7). **(D)** Confocal microscopy image of a sagittal brainstem slice (12 μm) showing immunolabeling of Kv3.3b K^+^ channels in the DCN molecular (M) and fusiform (F) layer (separated by a dashed line). Red arrow indicates example fluorescence punctum. **(E)** Same area is labeled with the pre-synaptic active zone protein Bassoon. **(D,E)** Insets show images of the molecular layer in which the primary antibody was omitted (scale bar = 20 μm). **(F)** Confocal image of the molecular layer showing the joint expression of Kv3.3 K^+^ channels (green) and Bassoon (red). Top right: Rectangular insets numbered 1 and 2 are *z*-axis representations of the images within yellow boxes (labeled 1 and 2). Bottom left: Line plot overlays show the fluorescence intensities (arbitrary units) for the green and red channels along the diagonal white line. **(G)** Mander’s R correlation coefficient showing the percentage of red fluorescence overlapping with green fluorescence for 12 fields from 3 slices (*N* = 2). **(H)** Image shown in panel **(F)** following filtering and fluorescence separation between green (Kv3.3) and red (Bassoon) objects, allowing for object-based co-localization (where yellow represents the overlap between green and red objects, estimated at 27.3% for this image). **(I)** Object-based co-localization for 12 fields from 3 slices (*N* = 2). **(G,I)** Error bars = SD. **p < 0.01.

We first checked that AUT1 modulated K^+^ currents mediated by the Kv3.1b and Kv3.3 K^+^ channel subtypes by expressing them in HEK293 cells and recording outward currents in the voltage-clamp recording configuration. Figures 5A–C, 6A–C show that AUT1 is indeed a positive modulator of Kv3.1b and Kv3.3 K^+^ channels, respectively. Kv3.1b K^+^ currents characteristically showed rapid activation and deactivation kinetics, very slow inactivation, and a voltage activation point of approximately −30 mV (Figures 5A,B). Similar to previous studies, 10 μM AUT1 shifted the voltage activation to more negative potentials as revealed by a leftward shift in the normalized conductance (G/G_*max*_) curve (Figure 5B, *n* = 7) and a significant decrease in the half-activation voltage (Figure 5C, V_50_ in control: −11.4 ± 5.6 mV; V_50_ in AUT1: −21.1 ± 6.3 mV, *n* = 7, paired *t*-test: *t*(6) = 4.9, *P* = 0.003) (Rosato-Siri et al., 2015; Brown et al., 2016). Similar results were also obtained using 30 μM AUT1 (V_50_ in control: −11.1 ± 5.0 mV; V_50_ in AUT1: −26.0 ± 11.0 mV, *n* = 5). Outward currents mediated by Kv3.3 K^+^ channels activated and deactivated rapidly, inactivated quickly by comparison to Kv3.1b K^+^ channels (Figure 6A), and activated at voltages of approximately −30 to 0 mV (Figures 6A–C). AUT1 (10 μM) shifted the voltage activation to more negative potentials, causing a leftward shift in the normalized conductance (G/G_*max*_) curve (Figure 6B, *n* = 7) and a significant decrease in the half-activation voltage (Figure 6C, V_50_ in control: 10.1 ± 7.3 mV; V_50_ in AUT1: 0.6 ± 10.9 mV, *n* = 7, paired *t*-test: *t*(6) = 3.9, *P* = 0.008). Similar results were also obtained using 30 μM AUT1 (V_50_ in control: 7.0 ± 2.8 mV; V_50_ in AUT1: −1.0 ± 4.8 mV, *n* = 3).

Kv3.1b K^+^ channel protein was weakly expressed in the DCN (Figures 5D,G–I), with negligible expression in the molecular layer (Figure 5G) and only weak staining on cells bodies in the fusiform (Figure 5H) and deep (Figure 5I) layers (yellow arrows). This was not due to a general lack of effect of the Kv3.1b antibody, as revealed by staining at the location medial to the DCN (Figures 5D,E, cyan arrows, cell bodies) and in the cerebellar granular cell layer (Figures 5D,F, magenta arrows). By contrast, immunolabeling against Kv3.3 K^+^ channels revealed a strong expression in the DCN, with punctate labeling clearly present in the molecular layer (Figure 6D, example punctum indicated by red arrow). This pattern of expression shows a high degree of similarity with the calcium fluorescence from the molecular layer evoked in response to stimulation of the parallel fibers (Figure 4G), suggesting that Kv3.3 K^+^ channels were localized at pre-synaptic boutons. Pre-synaptic puncta were labeled with Bassoon, a protein present in the active zone of pre-synaptic boutons (Dick et al., 2003; Al-Osta et al., 2018). Confocal images revealed a high density of fluorescent puncta labeled with Bassoon which was largely present in the molecular layer (Figure 6E, left of the white dashed line), a result reflecting the high density of parallel fiber boutons in the molecular layer (Salloum et al., 2014). The diameter of individual puncta labeled with Bassoon was 0.43 ± 0.20 μm (*N* = 3, *n* = 3, fields = 12, number of Bassoon puncta: 4319). The diameter of responding puncta in SyGCaMP2 mice was 0.84 ± 0.33 μm (Figures 4J,K, *N* = 3, *n* = 5, number of SyGCaMP2 fluorescence puncta: 393). These values are similar to the previously reported value of ∼0.5 μm for parallel fiber terminals in rodents (Tzounopoulos et al., 2007; Salloum et al., 2014). Figure 6F shows the overlap between green (Kv3.3) and red (Bassoon) fluorescence. The Manders’ R correlation coefficient quantifying the percentage of Bassoon co-localized with Kv3.3 K^+^ channels was 35.5 ± 9.8% (Figure 6G, *N* = 2, *n* = 3, fields = 12). An object-based co-localization (Figure 6H) led to a co-localization of 35.3 ± 8.4% (Figure 6I, *N* = 2, *n* = 3, fields = 12), confirming that the Manders’ R correlation coefficient reflected the number of individual boutons containing Kv3.3 K^+^ channels rather than a disproportionate clustering of the Kv3.3 K^+^ channels across boutons (for example, a very small number of boutons overlapping entirely with Kv3.3 K^+^ channels, or a very large number of boutons overlapping partially with Kv3.3 K^+^ channels). In summary, Kv3.3 K^+^ channels are present in about a third of pre-synaptic boutons within the DCN molecular layer.

### Effects of AUT1 on Axonal Kv3 K^+^ Currents

**FIGURE 7:**
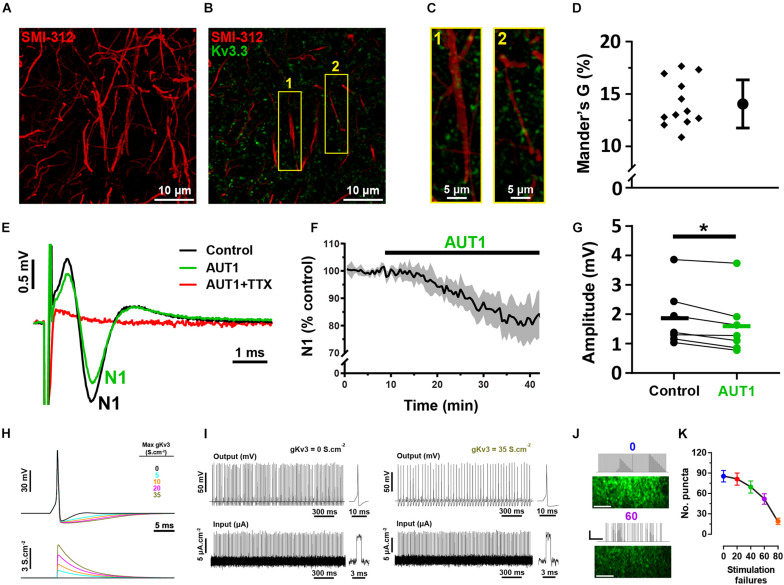
Immunolabeling and mathematical modeling reveal a moderate Kv3 mediated effect on bouton elimination via axonal action potential propagation. **(A)** Confocal image of axons in the molecular layer of the DCN (sagittal slice), labeled with the neurofilament marker SMI-312. Image shows the maximum fluorescence intensity from an entire z-stack (28 images taken at intervals of 45 nm). **(B)** Composite image showing the expression of Kv3.3 K^+^ channels (green) and the neurofilament marker SMI-312 (red) in the molecular layer. **(C)** 3D projection images (created using the volume viewer function in imageJ) from the yellow regions of interest labeled 1 and 2 in panel **(B)**. **(D)** Mander’s G correlation coefficient indicating the percentage of green (Kv3.3) fluorescence overlapping with axonal red (SMI-312) fluorescence (*N* = 2, *n* = 3, fields = 12). **(E)** Pre-synaptic fiber volleys (N1) obtained using extracellular recordings from the molecular layer in response to a 250 μA stimulation of the parallel fibers, in the control (black), AUT1 (green), and AUT1+TTX (red) conditions. **(F)** Time course for the effects of AUT1 on the amplitude of N1. Data are plotted as a percentage of control and represent the mean ± SEM (*n* = 6). **(G)** Perfusion of AUT1 significantly decreased the amplitude of N1 (*n* = 7). **(H)** Simulated single action potentials (top) represented as a function of varying Kv3 K^+^ conductance magnitudes (bottom). **(I)** Simulated action potentials (top) in response to 50 Hz stimulus trains (bottom), in absence (left, 0 S.cm^– 2^ gKv3) and in presence of a Kv3 K^+^ conductance (right, 35 S.cm^– 2^ gKv3). Insets represent simulated action potentials (top) elicited by single stimulus pulses (bottom). **(J)** SyGCaMP2 fluorescence responses obtained from the molecular-fusiform layer border in response to continuous 50 Hz stimulus train (top), or a 50 Hz stimulus train with 60 stimulus pulses omitted at random locations (bottom). Scale bars in fluorescence images = 20 μM. Stimulus trace horizontal and vertical scale bars = 400 ms and 50 μA, respectively. **(K)** Number of responding puncta as a function of the number of stimulation failures. Omitting stimulus pulses within the stimulus train significantly decreased the number of responding puncta (*n* = 7). Error bars = SEM. *p < 0.05.

Our results do not distinguish between an effect of Kv3 subunit containing K^+^ channels at the pre-synaptic terminal itself to inhibit calcium influx and transmitter release and an effect on the axons to prevent action potential propagation along pre-synaptic parallel fibers. To address this latter possibility, we measured the degree of co-localization between parallel fibers and Kv3.3 K^+^ channels using the antibody SMI-312, an axonal neurofilament marker (Figure 7A; Ulfig et al., 1998). Kv3.3 K^+^ channels were weakly co-localized with parallel fiber axons (Figures 7B,C), as revealed by an average Mander’s G correlation coefficient of 14.3 ± 2.1% (Figure 7D, *N* = 2, *n* = 3, 12 fields). Using brain slice recordings, AUT1 caused a significant decrease in the amplitude of the population action potential evoked in response to parallel fiber stimulation, otherwise known as the parallel fiber pre-synaptic fiber volley (N1), from 1.87 ± 1.0 mV to 1.61 ± 1.0 mV (Figures 7E–G, *n* = 7, *N* = 4, Wilcoxon signed-rank test: *W* = −28, *P* = 0.02), resulting in a percentage decrease of 16.4 ± 10.0%. These results indicate a small (∼16%) but non-negligible contribution of axonal Kv3 channels. Using a modeling approach, we next sought to determine whether this small effect on axonal Kv3 K^+^ currents contributes to the reduction of the number of responding pre-synaptic boutons observed during SyGCaMP2 fluorescence imaging experiments (Figures 4G–Q). As the potentiation of Kv3 K^+^ currents increases the action potential after-hyperpolarization (Chambers et al., 2017; Olsen et al., 2018), we hypothesized that this would result in action potential failure in response to a 50 Hz, 100 pulse stimulation (the same stimulation parameters used to measure the bouton fluorescence responses depicted in Figure 4). Action potential firing was modeled using a Hodgkin and Huxley based model (Hodgkin and Huxley, 1952), with an increase in Kv3 K^+^ conductance (from 0 to 35 S.cm^–2^) simulating the effects of AUT1 on the action potential waveform (Figure 7H), namely an increase in the size and duration of the action potential after-hyperpolarization, while failing to affect other action potential waveform properties (Chambers et al., 2017; Olsen et al., 2018). As predicted, increasing Kv3 K^+^ conductance increased action potential failure (Figure 7I). We then tested whether artificially increasing the number of action potential failures in brain slice recordings could reduce the number of responding pre-synaptic boutons (Figures 4G–Q). Introducing stimulation failures by omitting stimuli at random locations in the 50 Hz pulse train (Figure 7J) decreased the number of responding boutons (Figure 7K, *n* = 7, *N* = 4, Friedman test: χ^2^ = 23.9, *P* < 10^–3^). Therefore, our results suggest that a joint contribution of axonal and pre-synaptic Kv3.3 K^+^ channels contribute to a reduction in the number of responding boutons in presence of AUT1.

### Positive Modulation of Kv3 K^+^ Currents Decreases Action Potential Firing Frequency While Increasing the Coherence of Cross-Unit Spike Timing

**FIGURE 8:**
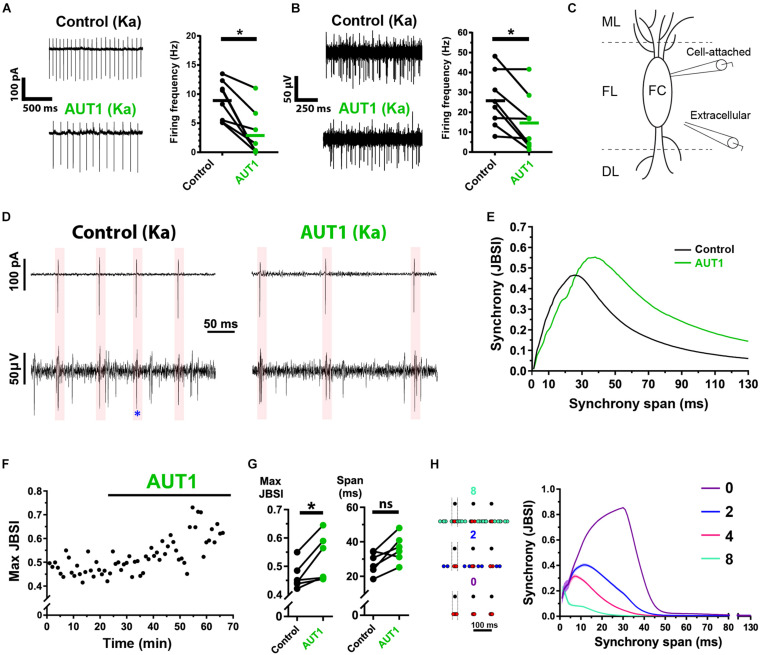
Positive modulation of Kv3 K^+^ currents increases local cross-unit synchrony of action potential firing in the fusiform layer of the dorsal cochlear nucleus. **(A)** Left: Example traces showing spontaneous action potential firing recorded from a fusiform cell using the cell-attached recording configuration, in the control (top) and AUT1 (bottom) conditions. Right: Perfusion of AUT1 decreased the firing frequency (*n* = 8). **(B)** Left: Example traces showing spontaneous action potential firing recorded from the fusiform layer using the extracellular recording configuration, in the control (top) and AUT1 (bottom) conditions. Right: Perfusion of AUT1 decreased the firing frequency (*n* = 8). **(C)** Schematic of the recording configuration allowing the measurement of cross-unit synchrony. ML, FL, and DL are the molecular, fusiform, and deep layer, respectively. FC = fusiform cell. **(D)** Simultaneous cell-attached recordings from a fusiform cell (top traces) and extracellular field potential recordings from the FL (bottom traces), in the control (left) and AUT1 (right) conditions. Shaded red areas allow visualizing cross-unit synchronous firing of action potentials. Blue asterisk in left (control) panel points to an action potential recorded in the cell-attached and extracellular recording configuration. **(E)** The jitter-based synchrony index (JBSI) is plotted as a function of synchrony span for an example brain slice recording in the control (black) and AUT1 (green) conditions. **(F)** The maximal peak of the JBSI curve is plotted as a function of experimental time for results depicted in panel **(E)**. **(G)** Perfusion of AUT1 increased the maximal JBSI (left, *n* = 6) but failed to affect the synchrony span at maximal JBSI (right, *n* = 6). **(H)** Left: Example portions of simulated cell-attached (reference, black dots, top rows) and extracellular (target, colored dots, bottom rows) spike times being either synchronous or asynchronous to each other. Numbers above the dots (0, 2, or 8) represent the number of asynchronous target spike times, represented as green and blue dots in the upper and middle rows, respectively. Target spike times occurring in phase with reference spike times are represented using red dots. Right: JBSI plotted as a function of synchrony span (ms) for simulated spike trains. Decreasing the number of asynchronous target spikes (number indicated in the inset) increases the maximal JBSI and shifts the JBSI versus synchrony span curve to the right. The time window in which synchronous target spike times occur with respect to a reference spike time is ±10 ms (illustrated by the vertical dashed lines surrounding the first reference spike times). Shaded error bars = SD. *p < 0.05.

Tinnitus is characterized by an increase in hyperexcitable action potential firing within multiple structures in the central auditory system, including the DCN (Kaltenbach, 2006; Wu et al., 2016). We therefore extended our study to measure the effect of Kv3 K^+^ channel positive modulation on spontaneous action potential firing and cross-unit synchrony, thus examining how synaptic noise regulation relates to network-level excitability. We first tested whether Kv3 K^+^ channel positive modulation affected the firing rate of individual principal fusiform cells and population firing in the fusiform layer, using cell-attached and extracellular recordings, respectively. DCN fusiform cells fire spontaneous action potentials in ∼50% of cases (Leao et al., 2012; Zugaib et al., 2016). We therefore added kainic acid to the perfusion bath to increase excitability before testing the effects of AUT1 (Cunningham et al., 2003). AUT1 reduces action potential firing in multiple structures within the central auditory system (Chambers et al., 2017; Glait et al., 2018). Kainic acid was therefore additionally used to reduce the probability of AUT1 eliminating all action potentials during an experiment, which in turn would preclude the comparison of action potential firing properties (e.g., synchrony) in the control and AUT1 conditions. Kainic acid (750 nM) increased the action potential firing frequency of fusiform cells recorded using the cell-attached recording configuration from 4.5 ± 3.3 Hz to 9.0 ± 3.3 Hz (*n* = 10, *N* = 6, paired *t*-test: *t*(9) = 6.3, *P* = 10^–4^). Similarly, kainic acid increased action potential firing frequency of cells within the fusiform layer recorded using the extracellular recording configuration from 17.6 ± 6.7 Hz to 24.3 ± 11.7 Hz (*n* = 10, *N* = 6, paired *t*-test: *t*(9) = 2.6, *P* = 0.027). Following the perfusion of kainic acid and the increased firing rates reaching steady-state, recordings in the presence of kainic acid were taken as the control condition for measuring the effects of AUT1 on spontaneous firing rates and action potential synchrony. Perfusion of AUT1 decreased the fusiform cell (cell-attached recording configuration) firing rate from 8.9 ± 3.8 Hz to 3.0 ± 4.0 Hz (Figure 8A, *n* = 8, *N* = 5, Wilcoxon signed rank test: *W* = −36, *P* = 0.016), and decreased the firing of neuronal populations in the fusiform layer (extracellular recording configuration) from 25.9 ± 13.8 Hz to 14.9 ± 14.2 Hz (Figure 8B, *n* = 8, *N* = 5, paired *t*-test: *t*(9) = 3.4, *P* = 0.023). Action potential amplitudes remained stable across the control and AUT1 conditions, for cell-attached (control: −144.1 ± 49.4 pA; AUT1: −153.2 ± 41.0 pA, paired *t*-test: *t*(6) = 1.4, *P* = 0.22) and extracellular (control: 60.7 ± 11.9 μV; AUT1: 56.8 ± 14.7 μV, paired *t*-test: *t*(5) = 0.67, *P* = 0.54) recordings, suggesting that the decrease in firing frequency in presence of AUT1 was not due to a reduction in action potential amplitude, which could limit the number of action potential detections above threshold. The coherence of spike-timing between principal fusiform cells (recorded in the cell-attached condition) and neighboring neurons (recorded extracellularly) (Figures 8C,D) was next quantified using the JBSI (Agmon, 2012). AUT1 increased the maximal value of local synchrony from 0.46 ± 0.05 to 0.53 ± 0.08 (Figures 8E–G left, *n* = 6, *N* = 3, paired *t*-test: *t*(5) = 3.3, *P* = 0.044), highlighting its ability to increase local synchrony between neighboring cells in the fusiform layer. We were unable to detect an effect of AUT1 on the time span of the maximum synchrony (Figures 8E–G, right, control: 27.9 ± 6.4 ms; AUT1: 36.1 ± 7.9 ms, *n* = 6, *N* = 3, paired *t*-test: *t*(5) = 2.9, *P* = 0.072). We hypothesized that the increase in synchrony arose from a preferential decrease in extracellular action potentials firing out of phase with the “reference” fusiform cells recorded using the cell-attached recording configuration. We demonstrated that this was indeed the case by simulating spike trains while controlling the temporal relationship between cell-attached reference spike times and neighboring target spike times in the fusiform layer recorded extracellularly (Figure 8H, left). A decrease of non-synchronous target spikes resulted in an increase and shift to the right of the synchrony (JBSI) versus synchrony span (ms) curve (Figure 8H, right), and reproduced the results observed in presence of AUT1 (Figure 8G).

## Discussion

Synaptic noise due to the spontaneous release of neurotransmitters is a fundamental component in action potential elicitation and transmission (Fatt and Katz, 1950; Faisal et al., 2008; Rusakov et al., 2020). The role of pre-synaptic ion channels in the modulation of synaptic noise and network-level excitability remains largely unexplored. Here we show the mechanisms by which a single type of ion channel (Kv3 K^+^ channels) localized to pre-synaptic boutons exert a powerful modulatory effect on synaptic noise and network-level excitability in an auditory brainstem circuit associated with the induction of tinnitus. Our study focused on the DCN because of hyperexcitability well characterized in that auditory structure (Kaltenbach, 2006; Wu et al., 2016). We chose to examine the effects of Kv3 K^+^ channels as they strongly modulate excitability within the DCN (Pilati et al., 2012; Glait et al., 2018; Olsen et al., 2018) and on the central auditory system in general (Song et al., 2005; Johnston et al., 2010; Chambers et al., 2017; Choudhury et al., 2020). Kv3 K^+^ channels have also got a therapeutic potential to mitigate hyperexcitability associated with tinnitus (Chambers et al., 2017; Anderson et al., 2018; Glait et al., 2018).

We previously showed that DCN principal fusiform cells fire action potentials with a close temporal relationship to sharp, local voltage maxima (putative EPSP peaks) contained within subthreshold voltage fluctuations (see also Street and Manis, 2007; Olsen et al., 2018), and that a positive modulation of Kv3 K^+^ currents prevents an increase in synaptic noise following either the non-selective inhibition of K^+^ channels or the induction of hearing impairments following acoustic over-exposure (Olsen et al., 2018). The first step in our study consisted in investigating the role of Kv3 K^+^ currents on synaptic noise in the DCN and the effect on the initiation of fusiform cell action potentials. Positive modulation of Kv3 K^+^ currents using AUT1 reduced the frequency of both sEPSCs and sIPSCs (Figure 1). Previous studies have shown inhibitory inputs to fusiform cells to be largely di-synaptic in nature (Doiron et al., 2011; Tang and Trussell, 2017). The elimination of sIPSCs following the perfusion of blockers of excitatory transmission confirmed these findings (Figures 1B,C) and illustrated that the main effect of AUT1 was primarily a reduction in the excitatory synaptic drive to fusiform cells. The next step consisted in studying the relationship between synaptic noise and the firing of fusiform cell action potentials. Synaptic noise showed a high degree of coherence with the initiation of fusiform cell action potentials (Figures 2A–D). Furthermore, coherence was highest for synaptic noise in the 20–40 Hz frequency band, which was in turn reduced by a positive modulation of Kv3 K^+^ currents (Figures 2G,H). These results thereby demonstrate a close link between synaptic noise, action potential initiation and a modulation of Kv3 K^+^ currents.

Parallel fibers projecting onto fusiform cells are characterized by a high density of synaptic boutons (Salloum et al., 2014). We therefore studied the role of Kv3 K^+^ currents on synaptic transmission evoked from parallel fiber stimulation in the DCN molecular layer. Our results revealed a reduction in the amplitude and number of responding boutons following the perfusion of AUT1. The lack of effect of AUT1 on the number of non-responding boutons is at first puzzling as the reduction in the number of responding boutons should increase the number of non-responding boutons. Spontaneous calcium events are detectable using the SyGCaMP2 calcium reporter (Dorostkar et al., 2010) and it is possible that the number of non-responding boutons decreases as a result of the reduction of the spontaneous activity following the perfusion of AUT1 (Figures 1, 8). Moreover, Tian et al. (2009) show a relationship between the number of spontaneous action potentials and the amplitude of GCaMP fluorescence. It is therefore possible that non-responding boutons (which are detected solely using basal calcium levels, and not stimulus-evoked fluorescence) may completely disappear (i.e., their fluorescence falling below detection thresholds) following a reduction of spontaneous activity. It should also be noted that bouton counts (Figure 4Q) were obtained from different fields of view in the control and AUT1 conditions (see section “Materials and Methods”), thus making it very unlikely that counts of boutons were obtained from the same population across conditions. Therefore, the number of non-responding boutons gained is unlikely to precisely match the number of responding boutons eliminated following the perfusion of AUT1.

Immunostaining revealed a punctate expression pattern of Kv3 K^+^ channels in the molecular layer, with a proportion of parallel fiber boutons (∼35%) co-localized with these Kv3 labeled puncta (Figure 6), and a small but non-negligible proportion of parallel fiber axons also co-localized with these Kv3 labeled puncta (Figure 7). Immunohistochemical labeling revealed that the Kv3 labeled boutons and axons were of the Kv3.3 rather than of Kv3.1 subtype (Figures 5, 6). The minimal amount of Kv3.1 staining on cell bodies within the DCN fusiform and deep layers is consistent with previous research on rodents (Perney and Kaczmarek, 1997; Rusznák et al., 2008), while the lack of staining in the DCN molecular layer contrasts with previous reports (Perney and Kaczmarek, 1997; Zettel et al., 2007). As expression of Kv3.1 increases during the post-natal development of rodents (Perney et al., 1992; Boda et al., 2012), the discrepancy in staining could be due to the younger age (P14–P21) of mice used in the current study.

The expression of Kv3.3 was punctate in the molecular layer, with Kv3.3 punctum co-localizing only partially with parallel fiber pre-synaptic boutons and axons. These results point to a sparsely localized distribution of Kv3.3 K^+^ channels being responsible for the highly efficient modulation of synaptic noise in the DCN, an effect similar to the one mediated by pre-synaptic Kv3 K^+^ channels located at hippocampal mossy fiber boutons (Alle et al., 2011; Rowan et al., 2016). In addition to the role of Kv3.3 K^+^ channels (Figures 6, 7), a contribution of other Kv3 K^+^ channel subtypes, namely Kv3.2 and/or Kv3.4 subtypes, cannot be ruled out. However, their contribution is likely to be negligible due to their weak expression in the auditory brainstem of mice (Choudhury et al., 2020). Figure 6 shows that a proportion of Kv3.3 K^+^ channels in the DCN molecular layer are not co-localized with the pre-synaptic marker Bassoon (boutons) and/or SMI-312 (axons). The question arises whether a mechanism additional to the potentiation of axonal and/or pre-synaptic Kv3 K^+^ currents contributes to the elimination of synaptic noise in this study. For example, there are inhibitory cartwheel and/or stellate cells located in the molecular layer which project onto fusiform cells (Apostolides and Trussell, 2014; Golding and Oertel, 1997), and these cells may be modulated by AUT1. Kv3.3 K^+^ channels are unlikely to be located at these two inhibitory neurons, as AUT1 reduced EPSC amplitudes in the control condition to an extent (59%, Figure 1H) similar to the one observed in presence of blockers of inhibitory synaptic transmission (57%, Figure 1J), indicating an effect on excitatory synaptic transmission onto fusiform cells.

Our study shows that potentiation of Kv3 K^+^ currents decreases the fusiform cell spontaneous firing rates and thus demonstrates the ability to reduce hyperexcitable action potential firing in the DCN, a key factor in the induction of tinnitus (Kaltenbach, 2006; Wu et al., 2016). This effect on the firing rate is likely to be due to an elimination of calcium influx at a subset of pre-synaptic boutons (Figure 4) and an increase in the amplitude and duration of the fusiform cell after-hyperpolarization (Figures 7H,I, and see Olsen et al., 2018).

We further show an increase in cross-unit synchrony following the potentiation of Kv3 K^+^ currents. It is of interest that tinnitus is correlated with increased spontaneous firing rates and increased action potential synchrony (Seki and Eggermont, 2003; Bauer et al., 2008; Wu et al., 2016), and that a potentiation of Kv3 K^+^ currents has previously been shown to decrease hyperexcitability associated with tinnitus (Chambers et al., 2017; Anderson et al., 2018; Glait et al., 2018). Our result showing an increase in synchrony following a potentiation of Kv3 K^+^ currents could therefore seem contradictory with respect to previous conclusions relating synchrony to tinnitus. However, it should be noted that the distance between recording electrodes in this study was relatively small (∼30–50 μm), indicating that our study likely captures local synchrony between neighboring neurons.

Our study demonstrates the ability for a spatially sparse, single ion channel subtype localized to pre-synaptic sites (boutons and axons) to dramatically alter levels of synaptic noise and synaptic excitability in the auditory brainstem. As local cell-to-cell communication becomes more isolated from neighboring areas (Figures 8E–H), larger network areas within the entire DCN could become uncoupled, thus having substantial implications for tinnitus related to hyperexcitability and general network synchrony (Wu et al., 2016).

In conclusion, dysfunctions or downregulations of voltage-gated Kv3 K^+^ channels can lead to neuronal network hyperexcitability, eventually causing tinnitus (Kaltenbach, 2006; Wu et al., 2016; Shore and Wu, 2019), or other disorders showing dysfunctions in excitability such as epilepsy (Nascimento and Andrade, 2016). The identification of the pre-synaptic effect of the Kv3 positive modulation in the uncoupling of local networks and the reduction of neuronal excitability provides an important insight into targeting those pathological excitability changes.

## Data Availability Statement

The raw data supporting the conclusions of this article will be made available by the authors, without undue reservation.

## Ethics Statement

The animal study was reviewed and approved by Animal Welfare Ethical Review Committee.

## Author Contributions

TO, AC, NP, CL, NH, and MH: experimental conception. TO, AC, MŠ, NP, and NH: data collection and analysis. TO and MH: study design and manuscript writing. All authors provided critical feedback and approved the final manuscript.

## Conflict of Interest

NP was employed by company Autifony Srl. CL was employed by company Autifony Therapeutics Limited. The remaining authors declare that the research was conducted in the absence of any commercial or financial relationships that could be construed as a potential conflict of interest.

## Publisher’s Note

All claims expressed in this article are solely those of the authors and do not necessarily represent those of their affiliated organizations, or those of the publisher, the editors and the reviewers. Any product that may be evaluated in this article, or claim that may be made by its manufacturer, is not guaranteed or endorsed by the publisher.
